# Electric field–assisted anion-π catalysis on carbon nanotubes in electrochemical microfluidic devices

**DOI:** 10.1126/sciadv.adj5502

**Published:** 2023-10-12

**Authors:** M. Ángeles Gutiérrez López, Rojan Ali, Mei-Ling Tan, Naomi Sakai, Thomas Wirth, Stefan Matile

**Affiliations:** ^1^ Department of Organic Chemistry, University of Geneva, Quai Ernest Ansermet 30, CH-1211 Geneva 4, Switzerland.; ^2^ School of Chemistry, Cardiff University, Park Place, Main Building, Cardiff CF10 3AT, UK.

## Abstract

The vision to control the charges migrating during reactions with external electric fields is attractive because of the promise of general catalysis, emergent properties, and programmable devices. Here, we explore this idea with anion-π catalysis, that is the stabilization of anionic transition states on aromatic surfaces. Catalyst activation by polarization of the aromatic system is most effective. This polarization is induced by electric fields. The use of electrochemical microfluidic reactors to polarize multiwalled carbon nanotubes as anion-π catalysts emerges as essential. These reactors provide access to high fields at low enough voltage to prevent electron transfer, afford meaningful effective catalyst/substrate ratios, and avoid interference from additional electrolytes. Under these conditions, the rate of pyrene-interfaced epoxide-opening ether cyclizations is linearly voltage-dependent at positive voltages and negligible at negative voltages. While electromicrofluidics have been conceived for redox chemistry, our results indicate that their use for supramolecular organocatalysis has the potential to noncovalently electrify organic synthesis in the broadest sense.

## Teaser

Supramolecular microfluidics systems assure applicability of electric field–induced catalysis to organic synthesis in practice.

## INTRODUCTION

**Fig. 1.**
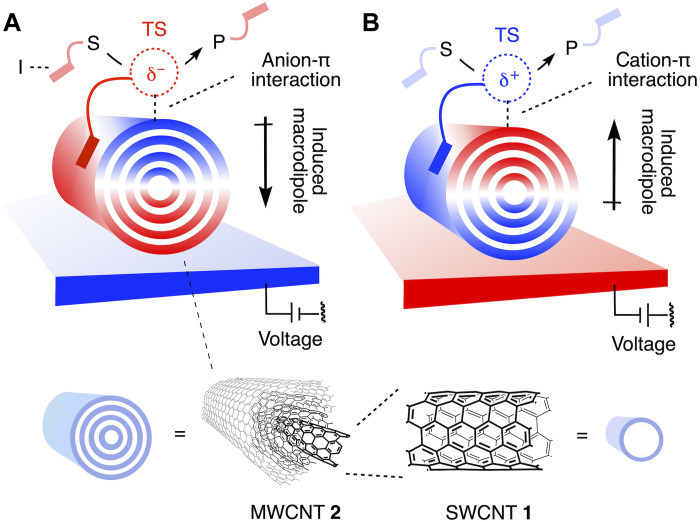
Systems design. Electric field–induced catalysis in electromicrofluidic reactors is realized with (**A**) anion-π and, by implication, (**B**) cation-π interactions on polarized multiwalled carbon nanotubes (MWCNTs) **2** to directionally stabilize anionic and cationic transition states (TS), respectively, and thus accelerate, at best modulate, the conversion of substrates (S) into products (P) (red, electron rich; and blue, electron poor), with interfacers (I) to assure substrate binding to the catalytic system.

Oriented external electric fields (OEEFs) hold much promise in theory to accelerate and direct the flow of charges during molecular transformations. Experimentally, such effects are of interest to accelerate chemical reactions in the broadest sense, enable different reactivities (*1*–*8*), and help understand and mimic enzyme function (*9*–*11*). Despite much effort and impressive single highlights, fundamental challenges have limited progress in organic synthesis in practice. Here, we show that anion-π catalysis (Fig. 1A) and, by implication, cation-π catalysis (Fig. 1B) on carbon nanotubes in electrochemical microfluidic reactors provide general access to electric field–induced catalysis under experimentally meaningful conditions.

Our approach combines emerging key principles from different directions. Electrochemical microfluidic reactors have been introduced recently to maximize the usefulness of electrochemical redox reactions in practice, including industrial and environmental aspects (*12*–*15*). Our study shows that the same electromicrofluidics used at voltages below the threshold of electron transfer can solve the central challenges with electric field–induced supramolecular organocatalysis. Namely, the short distance between the electrodes provides access to strong electric fields at the low voltages needed to avoid electron transfer. The close proximity of the electrodes also offers sufficient intrinsic conductivity to work without additional electrolytes, a major problem particularly for anion/cation-π catalysis in OEEFs. Moreover, the high ratio between electrode surface and solvent volume affords operational catalysis to substrate ratios at reasonably high substrate concentrations, and directional flow minimizes eventual product inhibition.

Composed of single-walled carbon nanotubes (SWCNTs) **1** wrapped around each other, multiwalled carbon nanotubes (MWCNTs) **2** are carbon allotropes of exceptionally high polarizability (Fig. 1). Electron relocation in response to charged molecules and OEEFs is possible not only along but also between the stacked, extended aromatic systems. This intra- and intertube electron relocation transforms OEEFs into giant oriented macrodipoles, producing oriented local electric fields that are strong enough for catalysis even at low external voltages below the onset of electron transfer. Depending on the orientation of the OEEF, these giant macrodipoles interact directionally with anionic and cationic parts of reactive intermediates and transition states to accelerate and direct the flow of electrons during the reaction. The result is anion-π and cation-π catalysis, respectively. Because the displacement of localized charge occurs during most reactions, electric field–induced anion/cation-π catalysis on MWCNTs in electrochemical microfluidic reactors is expected to be impactful in organic synthesis in the broadest sense.

**Fig. 2.**
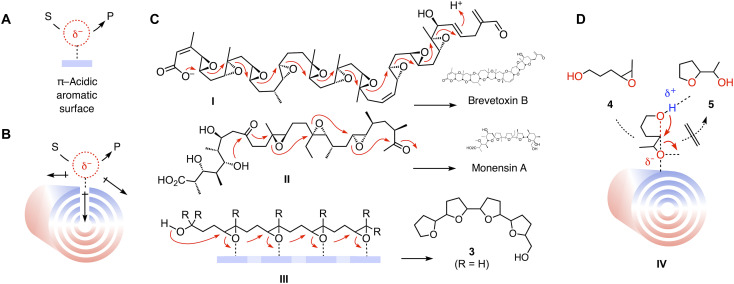
Anion-π catalysis. (**A**) General definition of anion-π catalysis. (**B**) Anion-π catalysis on MWCNTs **2**, from polarization by anionic transition states. (**C**) Epoxide-opening cascade cyclizations **I** to **III** leading to brevetoxin B and monensin A and on small-molecule anion-π catalysts (blue) in solution leading to oligo–tetrahydrofuran (THF) **3**. (**D**) Ether cyclization from **4** to **5**, with transition state **IV** for anion-π catalysis on MWCNTs, inaccessible without interfacing.

This study focuses on anion-π catalysis because the interactions of anions with π-acidic aromatic surfaces and their use in catalysis (*16*–*20*) are less developed than canonical cation-π interactions (*21*–*23*). Established in 2015 (*16*), anion-π catalysis is defined as the stabilization of anionic transition states on π-acidic aromatic surfaces (Fig. 2A). Induced anion-π interactions on larger aromatic systems with high polarizability have emerged as best for catalysis, from π-stacked foldamers (*24*) and fullerenes (*25*) to SWCNTs **1** as well as MWCNTs **2** with their supreme intra- and intertube polarizability (Fig. 2B) (*26*). Catalysis on carbon nanotubes (*27*–*38*) and graphene and graphite (*39*, *40*) has been explored quite extensively. The carbon allotropes are usually used as catch-and-release scaffolds and, in a few examples, as redox partners, including elegant regulation with stacked donors and acceptors (*31*), but explicitly neither for anion-π nor for cation-π catalysis beyond the example from this group (*26*). Although electric field–assisted anion-π interactions on graphene and graphite have been considered previously in theory (*41*–*44*), only one experimental proof-of-principle study exists from this group using a bifunctional catalyst of low polarizability (*45*).

The delocalized, polarizable yet directional nature of anion-π interactions suggests that, in catalysis, they would serve best to stabilize intermediates and transition states that involve charge displacement over long distances (*17*). This expectation is supported by steroid cyclization as the most spectacular expression of the charge inverted, conventional cation-π catalysis in nature, stabilizing the changing carbocation intermediates on π-basic amino acid side chains (*17*, *21*). Epoxide-opening polyether cascade cyclizations have been introduced recently as anionic counterpart of steroid cyclization for anion-π catalysis (*46*). They are best known from the Nakanishi hypothesis **I** for the biosynthesis of brevetoxin B and the Cane-Celmer-Westley hypothesis **II** for the biosynthesis of monensin A, exemplifying cascades that violate and follow the Eschenmoser-Dunitz-Baldwin guidelines, respectively (Fig. 2C) (*47*–*50*). On anion-π catalysts, cascade cyclization up to tetramer **III** has been realized. In the absence of directing methyl groups (*51*), the monensin A–like oligo–tetrahydrofuran (THF) product **3** is obtained (*46*).

Epoxide-opening ether cyclizations on π-acidic surfaces are interesting because they do not require additional activating groups and show autocatalytic behavior. This is true already for the cyclization of monomers such as **4** into THF **5** (*52*). Here, epoxide-opening ether cyclizations are used as benchmark reactions to elaborate on electric field–induced anion-π catalysis on carbon nanotubes in electrochemical microfluidic reactors.

## RESULTS

### Substrate interfacing

**Fig. 3.**
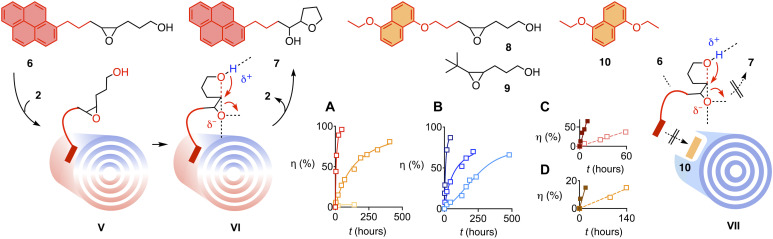
Catalysis by MWCNT suspensions. Structure of substrates (**6**, **8**, and **9**), products (**7**), and inhibitors (**10**), with notional catalyst-substrate complex **V**, transition state **VI**, and catalyst-inhibitor complex **VII**. (**A**) Conversion η of 100 mM **6** with time in the presence of 0 (yellow), 3 (orange) and 9 mol % **2** (red) in *ortho*-dichlorobenzene (ODCB) at 40°C. (**B**) Same for **8** with 1 (cyan), 3 (blue), and 9 mol % **2** (dark blue). (**C**) Same for **6** with 5 mol % **2**, with (empty, orange) and without (filled, grenat) 25 mol % **10**. (**D**) Same for **6** with 3 mol % **2**, with (empty, orange) and without (filled, grenat) 25 mol % **7**.

In suspension, MWCNTs **2** did not catalyze the conversion of substrate **4** substantially, i.e., transition state **IV** is inaccessible (Fig. 2D). To solve this problem, conjugate **6** was designed with an interfacer attached to the substrate (Figs. 1A and 3). The role of this interfacer is thought to assure binding of the substrate to the MWCNTs, which is to produce a formal substrate-catalyst complex **V**. The stability of this complex **V** should be sufficient to guarantee an effective concentration high enough for fast conversion into product **7**.

Pyrene is best known for interfacing with MWCNTs and also electrodes and used extensively for many purposes, including catalysis (*27*–*29*, *53*–*56*). The pyrene-substrate conjugate **6** was accessible from commercially available building blocks through a central, cis-selective Wittig reaction followed by epoxidation (Fig. 3). The dialkoxynaphthyl (DAN)–interfaced **8**, the *tert*-butyl control **9**, and inhibitor **10** were prepared analogously.

### Anion-π catalysis in MWCNT suspensions

The conversion of 100 mM of the pyrene-interfaced substrate **6** in *ortho*-dichlorobenzene (ODCB) at 40°C was monitored by high-performance liquid chromatography (HPLC) and nuclear magnetic resonance (NMR) spectroscopy. Rate enhancements *k*_cat_/*k*_uncat_ were obtained by dividing the measured rate constant *k*_cat_ of the catalyzed reaction by the measured rate constant *k*_uncat_ of the uncatalyzed reaction.The presence of 3 wt % MWCNTs **2** caused an enhancement *k*_cat_/*k*_uncat_ = 24 of the initial rate of substrate conversion (Fig. 3A, orange; and Table 1, entry 1). Already with 9 wt % **2**, the rate enhancement increased to *k*_cat_/*k*_uncat_ = 350, and conversion went to completion (Fig. 3A, red; and Table 1, entry 2). The rate of conversion of substrate **8** with DAN interfacers increased similarly in the presence of increasing amounts of MWCNTs (Fig. 3B and Table 1, entries 3 to 5). Very slow conversion of control substrate **9** with a hydrophobic *tert*-butyl in place of aromatic interfacers in the presence of 3 wt % **2** was detectable only at 60°C (Table 1, entry 6). These results supported the existence of anion-π catalysis of ether cyclizations on suspended MWCNTs and validated the importance of interfacers to bring substrate and catalyst in close proximity, better with pyrene than with DAN.

**Table 1.** Catalytic data for MWCNT suspensions. Conditions: 100 mM substrates (S), 1 to 9 wt % MWCNT catalyst, ODCB, 40°C (entry 6: 60°C), catalyst concentration, in wt %, rate enhancement, *k*_cat_ compared to *k*_ref_ (entries 1 to 6: *k*_ref_ = *k*_uncat_; entries 7 to 9: *k*_ref_ = *k*_i_ = *k*) with inhibitor at given condition, concentration of inhibitor (I) in mol % compared to S, and relation to structural interpretation (Int) in the figures.

Entry	S	MWCNT (wt %)	*k*_cat_/*k*_ref_	I	*c* (mol %)	Int
1	**6**	3	24	–	–	**VI**
2	**6**	9	350	–	–	**VI**
3	**8**	1	4	–	–	–
4	**8**	3	10	–	–	–
5	**8**	9	110	–	–	–
6	**9**	3	4	–	–	**IV**
7	**6**	5	11	**10**	25	**VII**
8	**8**	5	12	**10**	25	–
9	**6**	3	5	**7**	25	–

### Inhibition and autocatalysis

Already the presence of only 25 mol % DAN **10** decelerated anion-π catalysis with the pyrene-interfaced substrate **6** in MWCNT suspensions 11 times (Fig. 3C and Table 1, entry 7). Under the same conditions, anion-π catalysis with DAN interfacers **8** decreased to about the same extent (Table 1, entry 8). This similarity supported that inhibitor binding to the MWCNT accounts for decreasing activity by obstructing the binding of the substrates to the catalytic π surface, either physically, electrostatically, or both.

The kinetics of ether cyclizations catalyzed by MWCNTs **2** did not show autocatalytic behavior (Fig. 3, A and B). In the presence of autocatalysis, addition of 25 mol % product **7** at the beginning of the reaction would accelerate the conversion of substrate **6** on suspended MWCNTs (*52*). The fivefold deceleration found instead thus confirmed the presence of product inhibition caused by pyrene interfacers as outlined for **10** in **VII** (Fig. 3 and Table 1, entry 9). Rapid full conversion already at 9 wt % catalyst **2** confirmed that such product inhibition is insufficient to hinder multiple turnovers (Fig. 3A). One advantage of microfluidic reactors is that concerns of possible product inhibition essentially do not exist (vide infra).

The absence of anion-π autocatalysis in MWCNT suspensions further increased the importance of the observed rate enhancements, achieved without assistance from the product to an extent that is beyond reach with monomeric small-molecule anion-π catalysts (*52*). This maximized anion-π catalysis (Fig. 3A) coincided with maximized polarizability along and between carbon nanotubes (Fig. 2B) and thus suggested that the developed system could be well suited to amplify electric fields for anion-π catalysis in electromicrofluidic reactors (Fig. 1A).

### Electric field–assisted anion-π catalysis on MWCNTs

**Fig. 4.**
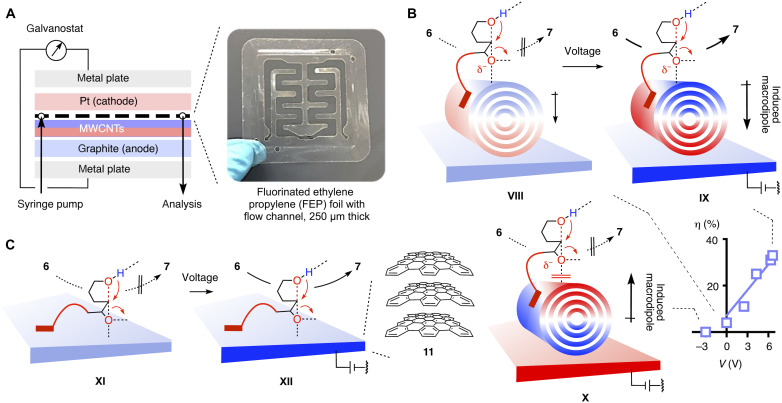
Catalysis in electrochemical microfluidic devices. (**A**) Electrochemical microfluidic reactor. (**B**) Conversion η of substrate **6** as a function of applied voltage on MWCNTs **2** on graphite electrodes in electromicrofluidic reactors [A; *Q*_v_ = 25 μl min^−1^, ODCB, room temperature (RT)], with notional mechanisms without (**VIII**), with activating (**IX**), and with inactivating voltage (**X**). (**C**) Notional mechanisms for the conversion of **6** on graphite electrodes **11** in electromicrofluidic reactors without (**XI**) and with activating voltage (**XII**).

The central piece of electromicrofluidic reactors is a thin fluorinated ethylene propylene (FEP) foil (Fig. 4A) (*12*). This foil is sandwiched between a graphite electrode, with or without MWCNT coating, and a platinum electrode, which are mounted between two metal plates. This foil is thin enough (250 μm) to allow current to flow without the addition of electrolytes. The assembled reactor is connected to a galvanostat and a syringe pump.

**Fig. 5.**
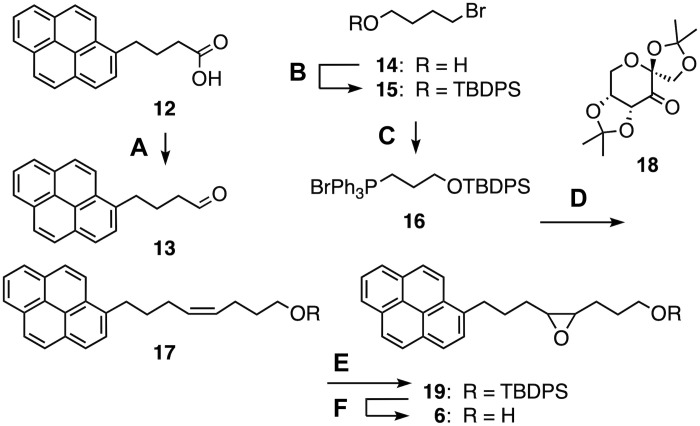
Synthesis of the key substrate. (**A**) (i) LiAlH_4_, THF, −78°C to RT, 15 hours, 74%; (ii) Dess-Martin periodinane (DMP), CH_2_Cl_2_, 0°C to RT, 2 hours, 54% (*61*, *62*); (**B**) 4-dimethylaminopyridine (DMAP), imidazole, *tert*-butyl(chloro)diphenylsilane (TBDPSCl), CH_2_Cl_2_, 0°C to RT, 3 hours, quant (*63*); (**C**) PPh_3_, toluene, 150°C, 15 hours, 65% (*52*); (**D**) (i) **16**, sodium bis(trimethylsilyl)amide (NaHMDS), THF, −78°C to 0°C, 20 min; (ii) **13**, −78°C to RT, 3 hours, 61%; (**E**) **18**, K_2_CO_3_, Na_2_B_4_O_7_/*n*-Bu_4_NHSO_4_ buffer, dimethoxymethane/MeCN 2:1, 0°C to RT, 15 hours, 66%; (**F**) tetrabutylammonium fluoride (TBAF), THF, 0°C to RT, 2 hours, 93%.

The electrode was coated by drop casting MWCNTs **2** on the freshly polished graphite electrode following literature procedures (*57*). Namely, a suspension of MWCNTs (2 mg ml^−1^) in *N*,*N*′-dimethylformamide was dropped on the electrode, air dried, rinsed with water, dried, and annealed at elevated temperature. The obtained electrode was assembled in the flow reactor, and substrate **6** was passed through the reactor with the syringe pump at a volumetric flow rate (*Q*_v_) = 25 μl min^−1^. Without applied current, substrate **6** was not converted under these conditions (Fig. 4B, **VIII**; and Table 2, entry 1). With increasing current applied, increasing conversion of substrate **6** was observed (Fig. 4B, **IX**; and Table 2, entries 2 to 6). At constant current applied by the galvanostat, the effective voltage usually varied within a small range of 0.2 V, often increasing slightly toward the end. Between different experiments, absolute voltage values could vary more, generally increasing, inter alia, with the age of the coated electrode. The plot of substrate conversion against the mean effective voltage showed quasi-linear dependence (Fig. 4B and Table 2). Inversion of the applied current up to −300 mA failed to turn on the reaction (Fig. 4B and Table 2, entry 7).

**Table 2.** Catalytic data for MWCNTs in electromicrofluidic reactors. Conditions: 250 mM substrate **6**, ODCB, RT, flow rate *Q*_v_ = 25 μl min^−1^ (entry 10: *Q*_v_ = 10 μl min^−1^), reactor configuration (anode/cathode), applied current, measured voltage range, measured substrate conversion, and relation to structural interpretation in the figures.

Entry	(+)/(−)	*I* (mA)	*V* (V)	η (%)	Int
1	MWCNT-Gr/Pt	0	0	4	**VIII**
2	MWCNT-Gr/Pt	100	2.4–2.6	11	**IX**
3	MWCNT-Gr/Pt	200	5.7	10	**IX**
4	MWCNT-Gr/Pt	300	5.8–6.9	31	**IX**
5	MWCNT-Gr/Pt	400	6.5–6.7	33	**IX**
6	MWCNT-Gr/Pt	600	4.2–4.3	25	**IX**
7	Pt/MWCNT-Gr	−300	−3.0	0	**X**
8	Gr/Pt	1	19–22	20	**XII**
9	Gr/Pt	3	24–29	45	**XII**
10	Gr/Pt	1	25–30	54	**XII**

The observed trends evinced that, without applied field, induced anion-π interactions are insufficient to effectively stabilize the transition state **VIII** under microfluidics conditions, while with increasing voltage, polarization of the MWCNTs induces powerful anion-π interactions to increasingly stabilize transition state **IX** and thus turn on anion-π catalysis (Fig. 4B). Together with unchanged products formed, quasi-linear dependence on the magnitude and on-off dependence on the direction of the applied field (i.e., independence of the current flowing) confirmed the occurrence of supramolecular, electric field–induced anion-π catalysis instead of redox processes from electron transfer.

### Electric field–assisted anion-π catalysis on graphite

Anion-π catalysis in electromicrofluidics on graphite **11** was more difficult to detect because, without MWCNTs, the conductivity in the flow reactor was much lower. This difference revealed that MWCNTs contribute much to the conductance of the system. At the much lower conductivity without MWCNTs, high voltages of 19 to 22 V were already generated by small currents of 1 mA (Table 2, entry 8). Under these conditions, conversion of substrate **6** was high. Conversion further increased when either the applied current was increased to 3 mA or the flow was reduced to 10 μl min^−1^ (Table 2, entries 9 and 10). Prolonged exposure to the graphite surface at least contributed to the latter besides effective voltage. As with MWCNTs, increased conversion upon increasing applied current provided proof-of-principle support that electric field–induced anion-π catalysis with interfaced substrates occurs also on graphite **11**. However, both conditions were not convincing because of either flow below microfluidics standards or very high voltage (24 to 29 V). Overall unchanged chemistry, not much dependent on the applied current, supported operational OEEF-induced catalysis without interference from electron transfer even at higher voltage on graphite. Nevertheless, high voltages are not desirable because of the risk of electron transfer and not needed in the present system because polarized MWCNTs amplify the applied OEEFs (Fig. 1A). Reducing the distance between the electrodes with thinner foils to increase the electric fields was not successful because of the occasional appearance of short circuits. Increasing the distance between the electrodes was not desirable because conductivity, electric fields (at constant voltage), and local catalyst to substrate ratios all decrease.

## DISCUSSION

This study provides a general method to enhance catalysis by OEEFs for general use in practice. It provides the tool needed to elaborate on high expectations from theory (*1*–*8*, *58*) and lessons from nature (*9*–*11*), enable research waiting to be realized, and, at best, transform organic synthesis in the broadest sense from control over chemo- and stereoselectivity to emergent properties, programmable multistep cascades, and, perhaps, even early steps in the possible origin of life (*59*, *60*).

The operational system combines electrochemical microfluidics and ion-π catalysis on carbon nanotubes. Electrochemical microfluidic reactors are crucial to overcome the need of additional electrolytes in conventional reactors, to maximize electric fields at low voltage and local catalyst to substrate ratios at reasonable substrate concentrations, and to minimize product inhibition. MWCNTs are important because their supreme polarizability amplifies OEEFs with local oriented macrodipoles that turn on the anion/cation-π interactions needed to accelerate and direct the movement of local charges during a reaction. Throughout the results, the occurrence of electric field–induced anion-π catalysis is supported by (i) quasi-linear dependence on the magnitude of the external electric field, (ii) on-off dependence on the direction of the applied field, (iii) quasi-independence on conductivity (i.e., the current flowing at given voltages), and (iv) unchanged product mixtures with and without electric fields. Electron transfer into the substrates would afford opposite characteristics: (i) superlinear dependence on the magnitude of the applied field, with sharp increases around the redox potential of the substrates; (ii) independence on the direction of the applied field; (iii) strong dependence on the applied current; and (iv) changed product mixtures with contributions from radical chemistry.

This practical access to electric field–induced catalysis has been realized by combining current key principles from different topics. The general nature of the results lifts each of these topics to another level of significance. With electrochemical microfluidic reactors, our results show that there is much to win before the electrons jump, shifting attention from radical to supramolecular chemistry, noncovalently electrifying organocatalysis. Anion-π catalysis and catalysis on carbon nanotubes both move up from specific to general, applicable, in principle, to any organic transformation.

Within each topic, there is much room for improvement. With anion-π catalysis confirmed, compatibility with cation-π catalysis should be easy to demonstrate (Fig. 1, A versus B). The interfacing of additional cocatalysts on the MWCNT surface will be most important to expand general applicability, particularly with regard to asymmetric catalysis. Concerning method development, most important will be interfacer engineering, up to traceless tags. Many possibilities exist to implement more advanced electrode surface modification methods and more elaborate electric and flow circuits for the remote control of multistep processes. Intense studies along these lines are ongoing and will be reported in due course.

## MATERIALS AND METHODS

### Materials

MWCNTs **2** were purchased from Sigma-Aldrich. Flow electrochemical experiments were performed using a stand-alone Vapourtec ion electrochemical reactor, with an Aim-TTi EX354RD dual power supply from Thurlby Thandar Instruments Ltd. A Chemyx Fusion 100 Touch syringe pump was used in the flow setup. Electrode materials used were platinum (Pt) and graphite (Gr) purchased from Goodfellow. The electrodes (5 cm by 5 cm) were separated by a 0.25-mm FEP spacer resulting in a reactor volume of 0.3 ml, with an exposed electrode surface area of 12 cm^2^. Full details on materials, synthetic methods, and instrumentation for chemical characterization can be found in the Supplementary Materials.

### Substrate synthesis

Full details on synthesis and characterization of all substrates (e.g., Fig. 5), products, and inhibitors can be found in the Supplementary Materials.

### Catalysis in suspension

Mixtures of substrates (**6**, **8**, **9**) and MWCNTs (**2**) were prepared in ODCB under an ambient atmosphere in a closed 1.5-ml glass vial and stirred at different temperatures. Aliquots (one drop, ~5 μl) of the mixture were taken at varying time intervals using a glass Pasteur pipette and diluted in CH_2_Cl_2_ or CD_2_Cl_2_ for analysis by HPLC or ^1^H NMR spectroscopy. Substrate conversion was determined either by comparing the area % of the pertinent peak (**6**: 5.48 min and **8**: 5.97 min) with that of the corresponding product (**7**: 2.40 min, product of **26**: 2.58 min) in the crude HPLC profile [YMC-Pack SIL, 120 Å; 3 μm, 0.8 ml/min, 10% (EtOAc + 1% Et_3_N) in CH_2_Cl_2_ for **6** and 30% (EtOAc + 1% Et_3_N) in *n*-hexane for **8**] or by comparing the integral of pertinent resonance [**9**: 2.65 parts per million (ppm)] with that of the corresponding product (**27**: 2.52 ppm) in the crude NMR spectra. In all cases, the starting material was converted selectively into THF [rather than tetrahydropyran (THP)] derivatives. Plots of substrate conversion against reaction time were recorded to determine the effects of catalyst loading, temperature, substrate, and inactivator concentrations.

### Catalysis in electrochemical microfluidic reactors

The reaction was performed in an undivided cell using a Vapourtec ion electrochemical reactor (FEP spacer, 0.25 mm; reactor volume, 0.3 ml; Pt, Gr, and MWCNT-coated Gr electrodes). The coating graphite electrode with MWCNTs was performed following an established drop-casting protocol (*57*). A solution of **6** (250 mM) in ODCB was pumped with different flow rates into the electrochemical reactor, and increasing current values were screened. The first one and a half reactor volumes (= 0.45 ml) were disposed to ensure that a steady state of the system had been reached. After collection for a defined period, the reaction mixture was analyzed by HPLC. The substrate conversion was determined by comparing the area % of pertinent peak (**6**: 4.13 min) with that of the corresponding product (**7**: 2.12 min) in the crude HPLC profile (Polaris Si, 250 mm by 4.6 mm by 5 μm; CH_2_Cl_2_/EtOAc/Et_3_N, 64:35:1; mobile phase, 2.2 ml/min). In all cases, the starting material was converted only into THF heterocycles as in **7**. Plots of substrate conversion against reaction time were recorded to determine dependence on voltage, current, flow rate, and concentration.
