# Supplementary material for: Electric field–assisted anion-π catalysis on carbon nanotubes in electrochemical microfluidic devices
Source: Sci Adv. 2023 Oct 12;9(41):eadj5502. doi: 10.1126/sciadv.adj5502 (PMC10569703; doi:10.1126/sciadv.adj5502)
Supplement: Supplementary file 1 — Supplementary Text Figs. S1 to S32 Table S1 [file sciadv.adj5502_sm.pdf]

Science Advances
AAAS

Supplementary Materials for

Electric field–assisted anion-π catalysis on carbon nanotubes in electrochemical microfluidic devices

M. Ángeles Gutiérrez López *et al.*

Corresponding author: Stefan Matile, stefan.matile@unige.ch

Sci. Adv. **9**, eadj5502 (2023)
DOI: 10.1126/sciadv.adj5502

This PDF file includes:

Supplementary Text
Figs. S1 to S32
Table S1

Supplementary Text

Materials

Reagents for synthesis were purchased from Merck, Apollo Scientific, Broadpharm, Sigma-Aldrich and Acros. Flash column chromatography was performed on a Biotage Isolera™ system. Analytical and preparative TLCs were performed on silica gel 60 F_{254} (Merck) and silica gel (SiliCycle, 1000 µm), respectively. Room temperature (RT) stands for 20-25 °C. Melting points (Mp) were measured on a Melting Point M-565 (BUCHI). IR spectra were recorded on a Perkin Elmer Spectrum One FT-IR spectrometer (ATR, Golden Gate) and are reported as wavenumbers ν in cm^{-1} with band intensities indicated as s (strong), m (medium), w (weak). ^{1}H and ^{13}C NMR were recorded (as indicated) either on a Bruker 300 MHz, 400 MHz, or 500 MHz spectrometer and are reported as chemical shifts (δ) in ppm relative to TMS (δ = 0). Spin multiplicities are reported as a singlet (s), doublet (d), triplet (t) and quartet (q), with coupling constants (J) given in Hz, or multiplet (m). Broad peaks are marked as br. ESI-MS was measured using Advion expression CMS and Advion plate express TLC/CMS, reported as *m/z*. Accurate mass determinations using ESI (HR ESI-MS) were performed on Xevo G2-S Tof (Waters). Flow electrochemical experiments were performed using a stand-alone Vapourtec Ion Electrochemical Reactor, with an Aim-TTi EX354RD Dual Power Supply from Thurlbym Thandar Instruments Ltd. Chemyx Fusion 100 Touch Syringe Pumps was used in the flow set-ups. Electrode materials employed were platinum (Pt) and graphite (Gr) purchased from Goodfellow. The electrodes (5 x 5 cm^2) were separated by a 0.25 mm FEP spacer resulting in a reactor volume of 0.3 mL, with an exposed electrode surface area of 12 cm^2.

Abbreviations

m-CPBA: meta-Chloroperoxybenzoic acid; DMAP: 4-Dimethylaminopyridine; DMP: Dess-Martin periodinane; EDTA: Ethylenediaminetetraacetic acid; LiHMDS: Lithium bis(trimethylsilyl)amide; MWCNT: Multi-walled carbon nanotube; NaHMDS: Sodium bis(trimethylsilyl)amide; ODCB: *o*-dichlorobenzene; RT: Room temperature; TBAF: Tetra-n-butylammonium fluoride; TBDPSCl: tert-Butyl(chloro)diphenylsilane.

Synthetic procedures

Compound **9** (*55*), intermediates **13** (*77*, *78*) and **16** (*55*) and inhibitor **27** (*55*) were prepared following published procedures.

Compound 15. Compound **14** (2.44 g, 12.8 mmol), DMAP (20 mg, 0.16 mmol) and imidazole (1.31 g, 19.3 mmol) were mixed in dry CH_2Cl_2 (18 mL) at 0 °C. The mixture was stirred for 10 minutes at this temperature and then TBDPSCl (5.0 mL, 19 mmol) was added dropwise. The mixture was warmed to RT and stirred for 3 h. The reaction mixture was filtered, the filtrate was concentrated under vacuum and purified by flash column chromatography (*n*-pentane/Et_2O 50:1, R_f 0.85) to obtain **15** (4.98 g, quant) as a colorless oil. The spectroscopic characteristics were consistent with reported values (*55*, *79*). R_f (*n*-pentane/CH_2Cl_2 10:1): 0.26; IR (neat): 3071 (w, C-H), 2932 (w, C-H), 2858 (w, C-H), 1427 (m, C-H), 1250 (w), 1104 (s, C-O), 822 (w), 699 (s, C-Br); ^{1}H NMR (400 MHz, CD_2Cl_2): 7.69 – 7.66 (m, 4H), 7.46 – 7.37 (m, 6H), 3.71 (t, ^{3}J = 6.5 Hz, 2H), 3.45 (t, ^{3}J = 6.5 Hz, 2H), 2.03 – 1.96 (m, 2H), 1.74 – 1.68 (m, 2H), 1.06 (s, 9H); ^{13}C NMR (126 MHz, $CDCl_3$): 135.9 (CH), 134.3 (C), 130.9 (CH), 127.5 (CH), 63.0 (CH_2), 34.1 (CH_2), 31.2 (CH_2), 29.6 (CH_2), 26.9 (CH_3), 19.4 (C); MS (ESI): 391 ($[M+H]^+$).

Compound 17. Compound **16** (1.92 g, 2.94 mmol) was dissolved in dry THF (15 mL) in an oven-dried Schlenk flask. The flask was evacuated and back-filled with nitrogen for 3 times. Then, the solution was cooled to –78 °C and NaHMDS solution in THF (1.0 M, 2.75 mL, 2.75 mmol) was added dropwise via syringe. The mixture was stirred at 0 °C for 20 min followed by the dropwise addition of **13** (0.50 g, 1.8 mmol) at –78 °C. The mixture was warmed up and stirred for 3 h at RT. The reaction mixture was quenched with saturated aqueous NH_4Cl (15 mL), and extracted with EtOAc (3 x 10 mL). The combined organic phases were dried over Na_2SO_4 and concentrated under vacuum. Further purification by flash column chromatography (*n*-pentane/EtOAc 9:1, R_f 0.77) gave **17** (0.64 g, 61%) as a colorless oil. R_f (*n*-pentane/EtOAc 24:1): 0.50; IR (neat): 3008 (w, C-H), 2930 (w, C-H), 1602 (w, C=C), 1432 (w, C-H), 1182 (w), 908 (s), 839 (s), 708 (m, C=C); ^{1}H NMR (500 MHz, $CDCl_3$): 8.26 (d, ^3J = 9.2 Hz, 1H), 8.16 (d, ^3J = 7.6 Hz, 1H), 8.10 (d, ^3J = 7.6 Hz, 1H), 8.08 (d, ^3J = 9.2 Hz, 1H), 8.03 (d, ^3J = 10.6 Hz, 1H), 8.01 (d, ^3J = 10.6 Hz, 1H), 7.99 (t, ^3J = 7.6 Hz, 1H), 7.87 (d, ^3J = 7.6 Hz, 1H), 7.73 – 7.63 (m, 4H), 7.43 – 7.31 (m, 6H), 5.52 – 5.42 (m, 2H), 3.69 (t, ^3J = 6.9 Hz, 2H), 3.33 (t, ^3J = 8.2 Hz, 2H), 2.26 – 2.22 (m, 2H), 2.20 – 2.16 (m, 2H), 1.94 – 1.88 (m, 2H), 1.67 – 1.62 (m, 2H), 1.07 (s, 9H); ^{13}C NMR (126 MHz, $CDCl_3$): 137.4 (C), 136.0 (CH), 134.5 (C), 131.8 (C), 131.3 (C), 130.5 (CH), 130.1 (C), 130.1 (CH), 129.9 (CH), 129.0 (C), 128.0 (CH), 127.9 (CH), 127.6 (CH), 127.5 (CH), 126.9 (CH), 126.2 (CH), 125.5 (C), 125.4 (C), 125.2 (CH), 125.0 (CH), 123.9 (CH), 63.8 (CH_2), 33.6 (CH_2), 33.1 (CH_2), 32.3 (CH_2), 27.7 (CH_2), 27.3 (CH_3), 24.1 (CH_2), 19.6 (C).

Compound 19. Compound **17** (82 mg, 140 µmol) was dissolved in dimethoxymethane/acetonitrile 2:1 (22.5 mL). A 0.05 M solution of $Na_2B_4O_7 \cdot 10\ H_2O$ (in 400 µM aqueous solution of Na_2EDTA, 15 mL), n-Bu_4NHSO_4 (6.0 mg, 17 µmol) and **18** (57 mg, 220 µmol) were sequentially added under vigorous stirring at 0 °C. To this mixture, a solution of oxone (109 mg, 649 µmol, in 400 µM aqueous solution of Na_2EDTA, 10.5 mL) and K_2CO_3 (0.36 g, 2.6 mmol, dissolved in water, 10.5 mL), were simultaneously added over 2 h via syringe pump. The mixture was stirred at RT for 15 h. At this point, the mixture was diluted with water (20 mL), extracted with CH_2Cl_2 (3 x 20 mL), washed with brine (2 x 20 mL), dried over Na_2SO_4, concentrated and purified by flash column chromatography (*n*-pentane/EtOAc 99:1 to 23:2) to give **19** (55 mg, 66%) as colorless oil. R_f (pentane/EtOAc 24:1): 0.23; IR (neat): 3044 (w, C-H), 2932 (w, C-H), 1738 (w, C-H), 1466 (w, C-H), 1241 (w, C-O-C), 1105 (s, C-O), 842 (m, C-O-C), 701 (s, C-O-C); ^{1}H NMR (500 MHz, $CDCl_3$): 8.27 (d, ^3J = 9.2 Hz, 1H), 8.16 (d, ^3J = 7.5 Hz, 2H), 8.11 (d, ^3J = 7.6 Hz, 1H), 8.09 (d, ^3J = 9.2 Hz, 1H), 8.02 (d, ^3J = 10.6 Hz, 1H), 8.01 (d, ^3J = 10.6 Hz, 1H), 7.99 (t, ^3J = 7.5 Hz, 1H), 7.87 (d, ^3J = 7.6 Hz, 1H), 7.68 – 7.66 (m, 4H), 7.47 – 7.29 (m, 6H), 3.75 – 3.64 (m, 2H), 3.52 – 3.27 (m, 2H), 3.02 – 2.97 (m, 1H), 2.97 – 2.88 (m, 1H), 2.16 – 1.94 (m, 2H), 1.79 – 1.67 (m, 4H), 1.66 – 1.62 (m, 2H), 1.06 (s, 9H); ^{13} C NMR (126 MHz, $CDCl_3$): 136.5 (C), 135.7 (CH), 134.1 (C), 134.0 (C), 131.6 (C), 131.0 (C), 130.0 (C), 129.8 (CH), 128.8 (C), 127.8 (CH), 127.7 (CH), 127.5 (CH), 127.4 (CH), 126.8 (CH), 126.0 (CH), 125.3 (C), 125.2 (C), 125.0 (CH), 125.0 (CH), 124.9 (CH), 123.4 (CH), 63.6 (CH_2), 57.2 (CH), 57.1 (CH), 33.5 (CH_2), 29.6 (CH_2), 29.0 (CH_2), 27.9 (CH_2), 27.0 (CH_3), 24.6 (CH_2), 19.4 (C); MS (ESI): 605 ($[M+Na]^+$).

Compound 6. Compound **19** (100 mg, 172 µmol) was dissolved in dry THF (3 mL), and a solution of TBAF in THF (1.0 M, 189 µL, 189 µmol) was added dropwise at 0 °C. After addition, the mixture was stirred for 2 h at RT. Then, the solvent was removed under vacuum and the crude was purified by flash column chromatography (*n*-pentane/Et_2O 7:3 to 0:1) to afford **6** (55 mg, 93%) as a colorless oil. R_f (*n*-pentane/Et_2O 3:7): 0.38; IR (neat): 3402 (w, O-H), 2933 (w, C-H), 2865 (w,

C-H), 1456 (m, C-H), 1181 (w, C-O-C), 1059 (m), 841(s, C-O-C); ^{1}H NMR (500 MHz, CD_2Cl_2): 8.32 (d, ^3J = 11.5 Hz, 1H), 8.19 (d, ^3J = 9.7 Hz, 1H), 8.18 (d, ^3J = 9.7 Hz, 1H), 8.15 (d, ^3J = 9.7 Hz, 1H), 8.13 (d, ^3J = 11.5 Hz, 1H), 8.04 (d, ^3J = 11.1 Hz, 1H), 8.03 (d, ^3J = 11.1 Hz, 1H), 8.01 (t, ^3J = 9.7 Hz, 1H), 7.92 (d, ^3J = 9.7 Hz, 1H), 3.61 (q, ^3J = 6.0 Hz, 2H), 3.51 – 3.35 (m, 2H), 3.03 – 2.97 (m, 1H), 2.97 – 2.90 (m, 1H), 2.14 – 1.94 (m, 2H), 1.75 – 1.58 (m, 5H), 1.49 – 1.45 (m, 1H); ^{13}C NMR (126 MHz, CD_2Cl_2): 137.1 (C), 131.8 (C), 131.3 (C), 130.2 (C), 129.0 (C), 127.9 (CH), 127.8 (CH), 127.6 (CH), 127.0 (CH), 126.3 (CH), 125.4 (C), 125.3 (C), 125.3 (CH), 125.2 (CH), 125.1 (CH), 123.8 (CH), 64.4 (CH_2), 57.5 (CH), 56.5 (CH), 34.7 (CH_2), 30.1 (CH_2), 29.2 (CH_2), 28.2 (CH_2), 26.0 (CH_2); HRMS (ESI): calcd. for $C_{24}H_{25}O_2$ ([M+H]$^+$): 345.1850, found: 345.1841.

Compound 7. Compound **6** (11 mg, 33 µmol) was dissolved in CH_2Cl_2 (0.1 mL), and $SbCl_3$ (0.8 mg, 3 µmol) was added. After addition, the mixture was stirred for 2 h at RT. Then, the reaction mixture was diluted with CH_2Cl_2 (1 mL) and washed with saturated aqueous NaOH (2 x 1 mL) and H_2O (1 x 1 ml). The organic phase was dried over Na_2SO_4 and concentrated under vacuum to give **7** (11 mg, 97%) as a colorless oil. R_f (Et_2O): 0.54; IR (neat): 3493 (s, O-H), 2928 (m, C-H), 2858 (m, C-H), 1404 (m, O-H), 1302 (m, C-H), 1060 (s, C-O), 833 (s, C-O); ^{1}H NMR (500 MHz, CD_2Cl_2): 8.33 (d, ^3J = 9.1 Hz, 1H), 8.18 (d, ^3J = 7.8 Hz, 1H), 8.17 (d, ^3J = 7.8 Hz, 1H), 8.13 (d, ^3J = 7.3 Hz, 1H), 8.12 (d, ^3J = 9.1 Hz, 1H), 8.05 (d, ^3J = 9.0 Hz, 1H), 8.03 (d, ^3J = 9.0 Hz, 1H), 8.00 (t, ^3J = 7.8 Hz, 1H), 7.91 (d, ^3J = 7.3 Hz, 1H), 3.82 – 3.71 (m, 2H), 3.68 – 3.66 (m, 1H), 3.49 – 3.42 (m, 1H), 3.43 – 3.32 (m, 2H), 2.36 (d, ^3J = 3.7 Hz, 1H), 2.16 – 2.03 (m, 1H), 2.00 – 1.92 (m, 1H), 1.92 – 1.81 (m, 3H), 1.66 – 1.51 (m, 4H); ^{13}C NMR (126 MHz, CD_2Cl_2): 137.6 (C), 131.8 (C), 131.4 (C), 130.1 (C), 129.0 (C), 127.9 (CH), 127.7 (CH), 127.5 (CH), 126.8 (CH), 126.2 (CH), 125.4 (C), 125.3 (C), 125.2 (CH), 125.0 (CH), 124.0 (CH), 82.9 (CH), 74.1 (CH), 68.4 (CH_2), 34.0 (CH_2), 33.8 (CH_2), 28.4 (CH_2), 28.3 (CH_2), 26.7 (CH_2); HRMS (ESI): calcd. for $C_{24}H_{25}O_2$ ([M+H]$^+$): 345.1850, found: 345.1841.

Compound 21. Compound **20** (7.0 g, 44 mmol) and K_2CO_3 (10.9 g, 78.7 mmol) were mixed in acetone (140 mL). Then, EtI (3.5 mL, 44 mmol) was added to the mixture. After addition, the mixture was stirred for 15 h at 60 °C. Then, 1 M HCl (30 mL) was added and the mixture was extracted with EtOAc (3 x 100 mL), washed with brine (2 x 100 mL), dried over Na_2SO_4 and concentrated under vacuum. Further purification by flash column chromatography (*n*-pentane/EtOAc 97:3 to 90:10; *n*-pentane/EtOAc 90:10 R_f 0.50) gave **21** (3.38 g, 41%) as a yellow solid. R_f (CH_2Cl_2/*n*-pentane 2:1): 0.38; Mp: 104 – 105 °C; IR (neat): 3234 (m, O-H), 2877 (w, C-H) 1595 (m), 1366 (s, O-H), 1249 (m, C-O), 1050 (s), 951 (m), 764 (s); ^{1}H NMR (400 MHz, CD_2Cl_2): 7.86 (d, ^3J = 8.6 Hz, 1H), 7.70 (d, ^3J = 8.6 Hz, 1H), 7.38 (t, ^3J = 8.6 Hz, 1H), 7.29 (t, ^3J = 8.6 Hz, 1H), 6.86 (d, ^3J = 8.6 Hz, 2H), 5.42 (s, 1H), 4.20 (q, ^3J = 7.0 Hz, 2H), 1.53 (t, ^3J = 7.0 Hz, 3H); ^{13}C NMR (101 MHz, CD_2Cl_2): 154.7 (C), 151.2 (C), 127.0 (C), 125.3 (CH), 125.3 (C), 125.0 (C), 114.6 (CH), 113.3 (CH), 109.3 (CH), 105.3 (CH), 63.9 (CH_2), 16.8 (CH_3); MS (ESI): 189 ([M+H]$^+$).

Compound 22. Compound **21** (3.38 g, 18.0 mmol) and K_2CO_3 (3.95 g, 28.6 mmol) were mixed in acetone (14 mL) and DMF (14 mL). Then, **14** (3.60 mL, 19.0 mmol) was added, and the mixture was stirred for 15 h at 80 °C. After that time the solid in suspension was filtered off and the filtrate was concentrated under vacuum. The residue was extracted with EtOAc (3 x 20 mL) and washed with brine (3 x 20 mL). The combined organic phases were dried over Na_2SO_4 and concentrated under vacuum. Further purification by flash column chromatography (CH_2Cl_2, R_f 0.20) gave **22**

(2.25 g, 48%) as a yellow solid. R_f (n-pentane/EtOAc 10:1): 0.49; Mp: 100 – 101 °C; IR (neat): 3495 (w, O-H), 2930 (w, C-H), 1508 (w), 1383 (m, O-H), 1260 (m, C-O), 1055 (s), 836 (m), 777 (s); ^{1}H NMR (500 MHz, CD_2Cl_2): 7.85 – 7.79 (m, 2H), 7.35 (t, ^3J = 7.6 Hz, 2H), 6.85 (dd, ^3J = 7.6, ^4J = 2.2 Hz, 2H), 4.19 (q, ^3J = 6.9 Hz, 2H), 4.17 (t, ^3J = 6.1 Hz, 2H), 3.73 (q, ^3J = 6.1 Hz, 2H), 2.03 – 1.96 (m, 2H), 1.86 – 1.79 (m, 2H), 1.53 (t, ^3J = 6.9 Hz, 3H); ^{13}C NMR (126 MHz, CD_2Cl_2): 155.0 (C), 155.0 (C), 127.1 (2C), 125.5 (CH), 125.4 (CH), 114.4 (CH), 114.3 (CH), 105.7 (CH), 105.7 (CH), 68.4 (CH_2), 64.3 (CH_2), 63.0 (CH_2), 30.1 (CH_2), 26.3 (CH_2), 15.0 (CH_3); MS (ESI): 283 ($[M+Na]^+$).

Compound 23. DMP (2.15 g, 5.07 mmol) was added portion-wise to a solution of **22** (1.11 g, 4.23 mmol) in CH_2Cl_2 (55 mL) at 0 °C. The mixture was stirred for 2 h at RT. Then, the mixture was diluted with CH_2Cl_2 (30 mL) and quenched with sat. $NaHCO_3$ (20 mL). The organic phase was washed with $Na_2S_2O_3$ (2 x 20 mL) and brine (2 x 20 mL). The crude product was purified by flash column chromatography (CH_2Cl_2/MeOH 47:3, R_f 0.77) to give **23** (0.86 g, 79%) as a yellowish solid. R_f (*n*-pentane/EtOAc 8:1): 0.73; Mp: 92 – 93 °C; IR (neat): 3503 (w), 2937 (w, C-H), 1715 (m, C=O), 1509 (w), 1388 (m, C=O), 1265 (s, C-O), 1059 (s), 778 (s), 613 (w); ^{1}H NMR (500 MHz, CD_2Cl_2): 9.87 (t, ^3J = 1.4 Hz, 1H), 7.84 (d, ^3J = 9.1 Hz, 1H), 7.77 (d, ^3J = 9.1 Hz, 1H), 7.39 – 7.32 (m, 2H), 6.85 (t, ^3J = 7.0 Hz, 2H), 4.19 (q, ^3J = 7.2 Hz, 2H), 4.17 (t, ^3J = 6.4 Hz, 2H), 2.74 (td, ^3J = 7.2 Hz, ^4J = 1.4 Hz 2H), 2.28 – 2.21 (m, 2H), 1.57 – 1.49 (m, 3H); ^{13}C NMR (126 MHz, CD_2Cl_2): 202.1 (CO), 155.8 (C), 154.7 (C), 127.1 (C), 127.0 (C), 125.6 (CH), 125.4 (CH), 114.6 (CH), 113.9 (CH), 105.8 (CH), 105.7 (CH), 67.5 (CH_2), 64.1 (CH_2), 41.4 (CH_2), 23.1 (CH_2), 15.2 (CH_3); MS (ESI): 260 ($[M+H]^+$).

Compound 24. Compound **16** (2.08 g, 3.18 mmol) was dissolved in dry THF (34 mL) in an oven-dried Schlenk flask. The flask was evacuated and back-filled with nitrogen for 3 times. Then, the solution was cooled to –78 °C and LiHMDS solution in THF (1.0 M, 6.6 mL, 6.6 mmol) was added via syringe dropwise. The mixture was stirred at RT for 30 min followed by the addition of **23** (0.68 g, 2.7 mmol) dropwise at – 78 °C. The mixture was warmed up and stirred for 4 h at RT. The reaction mixture was quenched with saturated aqueous NH_4Cl (20 mL) and the organic phase was extracted with EtOAc (3 x 20 mL). The combined organic phases were dried over Na_2SO_4 and concentrated under vacuum. Further purification by flash column chromatography (*n*-pentane/CH_2Cl_2 8:2, R_f 0.43) gave **24** (0.73 g, 50%) as a colorless oil. R_f (*n*-pentane/EtOAc 8:1): 0.69; IR (neat): 3070 (w, C-H), 2932 (w, C-H), 1593 (m, C=C), 1417 (m), 1387 (m), 1264 (s, C-O), 1077 (s), 1064 (s), 703 (s, C=C); ^{1}H NMR (500 MHz, CD_2Cl_2): 7.81 (d, ^3J = 10.3 Hz, 2H), 7.68 – 7.62 (m, 4H), 7.43 – 7.34 (m, 6H), 7.34 – 7.27 (m, 2H), 6.82 (d, ^3J = 9.8 Hz, 2H), 5.52 – 5.38 (m, 2H), 4.18 (q, ^3J = 8.5 Hz, 2H), 4.10 (t, ^3J = 8.2 Hz, 2H), 3.62 (t, ^3J = 8.4 Hz, 2H), 2.36 – 2.29 (m, 2H), 2.19 – 2.12 (m, 2H), 2.01 – 1.91 (m, 2H), 1.63 – 1.56 (m, 2H), 1.53 (t, ^3J = 8.5 Hz, 6H), 1.02 (s, 9H); ^{13}C NMR (126 MHz, CD_2Cl_2): 155.0 (C), 154.9 (C), 135.9 (CH), 134.5 (CH), 130.7 (C), 129.9 (CH), 129.4 (C), 128.0 (CH), 127.1 (C), 127.1 (C), 125.5 (CH), 125.4 (CH), 115.0 (CH), 114.4 (CH), 105.7 (C), 105.6 (C), 67.9 (CH_2), 65.1 (CH_2), 63.7 (CH_2), 33.1 (CH_2), 29.6 (CH_2), 27.0 (CH_3), 24.7 (CH_2), 23.9 (CH_2), 19.4 (C), 15.1 (CH_3); MS (ESI): 575 ($[M+Na]^+$).

Compound 25. Compound **21** (380 mg, 0.65 mmol) was dissolved in dry CH_2Cl_2 (22 mL) and *m*-CPBA (200 mg, 0.78 mmol) was added portionwise at 0 °C. After addition the mixture was stirred for 15 h at RT. The reaction mixture was concentrated under vacuum and purified by flash column chromatography (*n*-pentane/Et_2O 7:3, R_f 0.24) to give **25** (0.23 g, 58%) as a colorless oil. R_f (*n*-pentane/CH_2Cl_2 6:1): 0.22; IR (neat): 2931 (m, C-H), 2859 (m, C-H), 1593 (m), 1417 (m), 1263

(s, C-O-C), 1107 (s, C-O-C), 772 (s, C-O-C); ^{1}H NMR (400 MHz, CD_2Cl_2): 7.81 (d, ^3J = 10.7 Hz, 2H), 7.65 (dd, ^3J = 9.6, ^4J = 2.1 Hz, 4H), 7.44 – 7.34 (m, 6H), 7.34 – 7.28 (m, 2H), 6.86 (d, ^3J = 4.7 Hz, 2H), 6.83 (d, ^3J = 4.7 Hz, 2H), 4.21 – 4.15 (m, 4H), 3.76 – 3.66 (m, 2H), 3.03 – 2.97 (m, 1H), 2.96 – 2.90 (m, 1H), 2.15 – 2.01 (m, 2H), 1.87 – 1.66 (m, 4H), 1.68 – 1.59 (m, 2H), 1.54 (t, ^3J = 8.8 Hz, 6H), 1.03 (s, 9H); ^{13}C NMR (126 MHz, CD_2Cl_2): 154.6 (C), 154.5 (C), 135.5 (CH), 133.9 (C), 129.5 (CH), 127.6 (CH), 126.7 (C), 125.1 (CH), 125.0 (CH), 114.1 (CH), 113.9 (CH), 105.3 (CH), 105.3 (CH), 67.7 (CH2), 63.8 (CH_2), 63.5 (CH_2), 56.8 (CH), 56.6 (CH), 29.6 (CH_2), 26.6 (CH_3), 26.5 (CH_2), 24.8 (CH_2), 24.4 (CH_2), 19.0 (CH_2), 14.6 (CH_3); MS (ESI): 591 ($[M+Na]^+$).

Compound 8. Compound **25** (119 mg, 203 µmol) was dissolved in dry THF (7 mL) and TBAF solution in THF (1.0 M, 224 µL, 224 µmol) was added dropwise at 0 °C. After addition the mixture was stirred for 4 h at RT. Then, the solvent was removed under vacuum and the crude purified by flash column chromatography (*n*-pentane/Et_2O 7:3 to 0:1, R_f 0.43) to afford **8** (58 mg, 82%) as a colorless oil. R_f (Et_2O): 0.15; IR (neat): 3395 (m, O-H), 2933 (m, C-H), 2872 (w, C-H), 1593 (m), 1509 (m), 1415 (s), 1386 (s, O-H), 1263 (s, C-O-C), 1061 (s, C-O-C), 771 (s, C-O-C); ^{1}H NMR (500 MHz, CD_2Cl_2): 7.83 (dt, ^3J = 8.2, ^4J = 1.0 Hz, 1H), 7.81 (dt, ^3J = 8.2, ^4J = 1.0 Hz, 1H), 7.35 (t, ^3J = 8.2 Hz, 2H),, 2H), 6.85 (dd, ^3J = 8.2, ^4J = 1.0 Hz, 2H), 4.24 – 4.15 (m, 4H), 3.61 (q, ^3J = 6.2 Hz, 2H), 3.07 – 3.02 (m, 1H), 2.98 – 2.93 (m, 1H), 2.16 – 2.03 (m, 2H), 1.90 – 1.74 (m, 2H), 1.73 – 1.62 (m, 3H), 1.53 (t, ^3J = 7.0 Hz, 3H); ^{13}C NMR (126 MHz, CD_2Cl_2): 155.0 (C), 154.9 (C), 127.1 (C), 127.1 (C), 125.6 (CH), 125.5 (CH), 124.5 (CH), 114.5 (CH), 114.2 (CH), 105.8 (CH), 105.7 (CH), 68.0 (CH_2), 64.3 (CH_2), 62.7 (CH_2), 57.3 (CH), 30.2 (CH_2), 26.9 (CH_2), 25.2 (CH_2), 24.8 (CH_2), 15.0 (CH_3); HRMS (ESI): calcd. for $C_{20}H_{26}O_4$ ($[M+Na]^+$): 353.1724, found: 353.1709.

Compound 26. Compound **8** (40 mg, 115 µmol) was dissolved in CH_2Cl_2 (0.5 mL), and $SbCl_3$ (10 mg, 44 µmol) was added. After addition, the mixture was stirred for 2 h at RT. Then, the reaction mixture was diluted with CH_2Cl_2 (1.00 mL) and washed with saturated aqueous NaOH (2 x 1.00 mL) and H_2O (1 x 1.00 ml). The organic phase was dried over Na_2SO_4 and concentrated under vacuum to give **26** (37 mg, 97%) as a colorless solid. R_f (pure Et_2O): 0.74; Mp: 76 –77 °C; IR (neat): 3488 (m, O-H), 2973 (m, C-H), 2869 (m, C-H), 1417 (m), 1387 (s, O-H), 1266 (s, C-O), 1055 (s, C-O-C), 774 (s, C-O-C); ^{1}H NMR (400 MHz, CD_2Cl_2): 7.82 (dd, ^3J = 8.1, ^4J = 2.9 Hz, 2H), 7.35 (t, ^3J = 8.1 Hz, 2H), 6.85 (dd, ^3J = 8.1, ^4J = 2.9 Hz, 2H), 4.22 – 4.14 (m, 4H), 3.84 – 3.70 (m, 3H), 3.51 – 3.46 (m, 1H), 2.44 (s, 1H), 2.21 – 2.10 (m, 1H), 2.06 – 1.96 (m, 1H), 1.94 – 1.86 (m, 3H), 1.79 – 1.71 (m, 1H), 1.68 – 1.58 (m, 2H), 1.53 (t, ^3J = 7.3 Hz, 3H); ^{13}C NMR (101 MHz, CD_2Cl_2): 155.0 (C), 155.0 (C), 127.1 (CH), 127.1 (CH), 125.5 (CH), 125.5 (CH), 114.4 (CH), 114.3 (CH), 105.7 (CH), 82.9 (CH), 74.0 (CH), 68.5 (CH_2), 68.4 (CH_2), 64.2 (CH_2), 30.7 (CH_2), 28.3 (CH_2), 26.7 (CH_2), 26.1 (CH_2), 15.1 (CH_3); HRMS (ESI): calcd. for $C_{24}H_{24}O_2$ ($[M+Na]^+$): 353.1724, found: 353.1709.

Kinetics

Procedures for catalytic reactions in MWCNT suspensions and in electromicrofluidic reactors are described in the manuscript, Material and Methods part. To determine rate constants, the concentration of consumed substrate (**6**, **8** or **9)** was plotted against reaction time, and the initial velocities were determined from linear fitting (Fig. S30-S32). Apparent initial first-order rate constants (Table 1) were determined from Equation (S1)

$$k_{app} = v_{ini} / ([\text{epoxide}]_0) \qquad \text{(S1)}$$

where k_{app} corresponds to the first-order catalytic rate constant (k_{cat}) in the presence of MWCNTs and to the first-order rate constant of the uncatalyzed reaction (k_{uncat}). Constants k_{uncat} (**6**) = 2.3 x 10^{-5} h^{-1}, k_{uncat} (**8**) = 3.8 x 10^{-4} h^{-1} and k_{uncat} (**9**) = 2.7 x 10^{-2} h^{-1}.

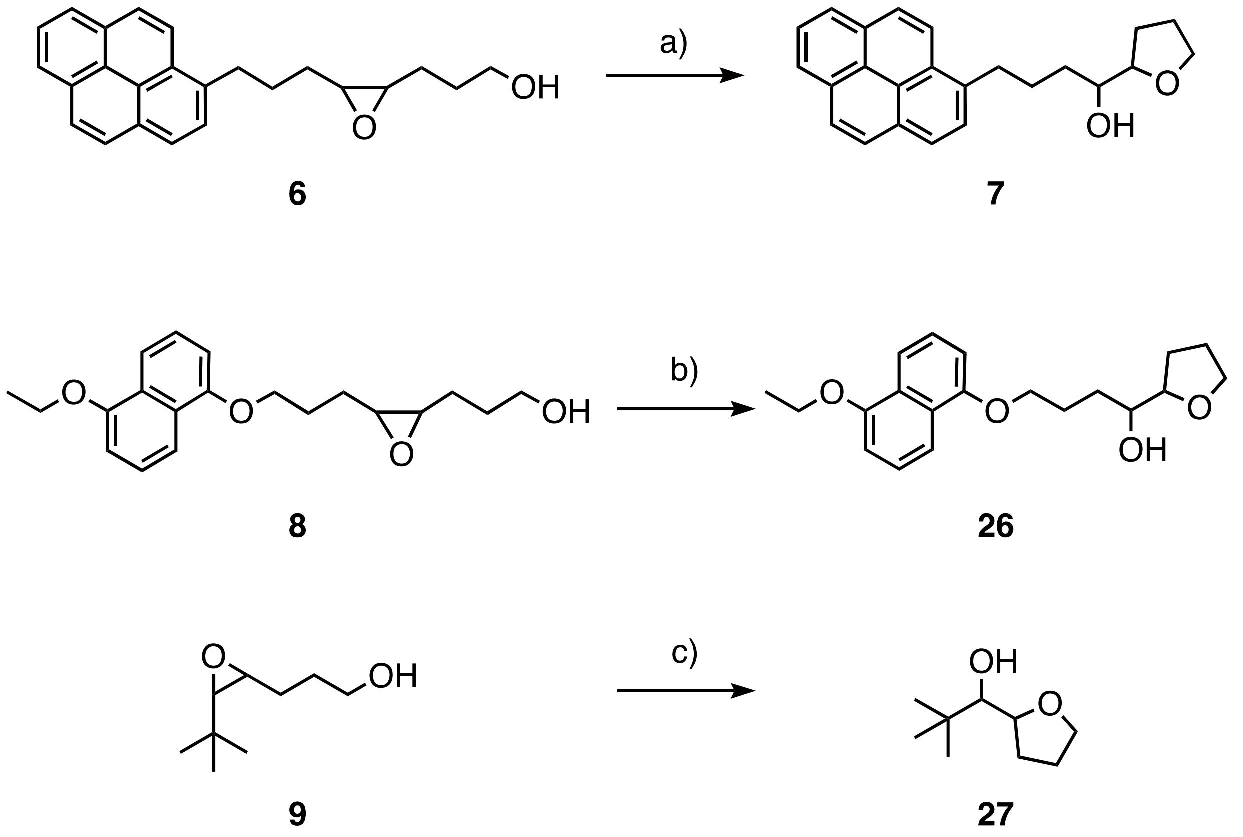

Fig. S1. Synthetic route for inhibitors 7, 26 and 27.

a) $SbCl_3$, CH_2Cl_2, RT, 2 h, 97%; b) $SbCl_3$, CH_2Cl_2, RT, 2 h, 97%; c) Synthesis and full characterization have been previously reported (*55*).

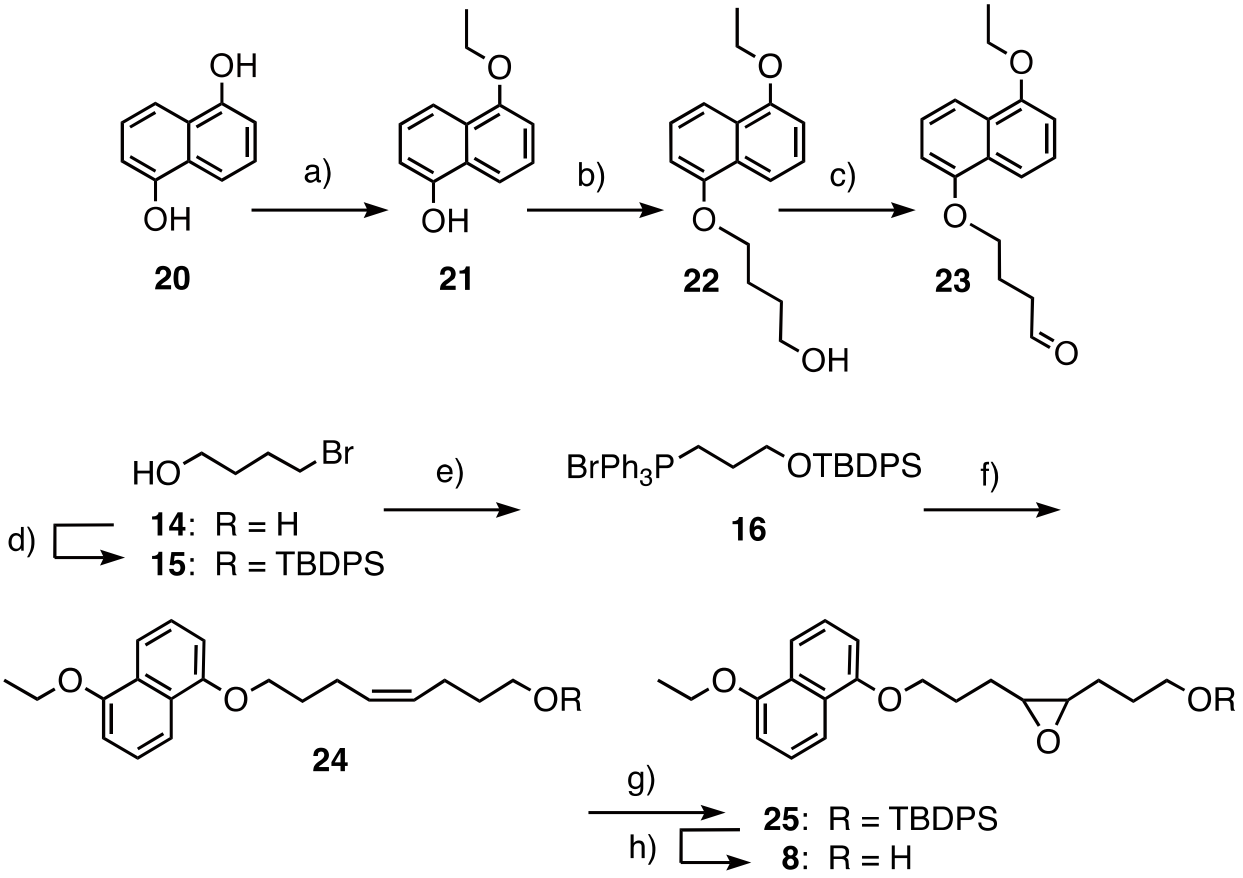

Fig. S2. Synthesis of DAN-interfaced substrate.

a) EtI, K_2CO_3, acetone, 60 °C, 15 h. 41%; b) **14**, K_2CO_3, acetone/DMF 1:1, 80 °C, 15 h, 48%; c) DMP, CH_2Cl_2, 0 °C to RT, 2 h, 79%; d) DMAP, imidazole, TBDPSCl, CH_2Cl_2, 0 ºC to RT, 3 h, quant; e) PPh_3, toluene, 150 °C, 15 h, 65%; f) 1. LiHMDS, THF, -78 °C to RT, 30 min; 2. **23**, -78 °C to RT, 4 h, 50%; g) *m*-CPBA, CH_2Cl_2, 0 °C to RT, 15 h, 58%; h) TBAF, THF, 0 °C to RT, 4 h, 82%.

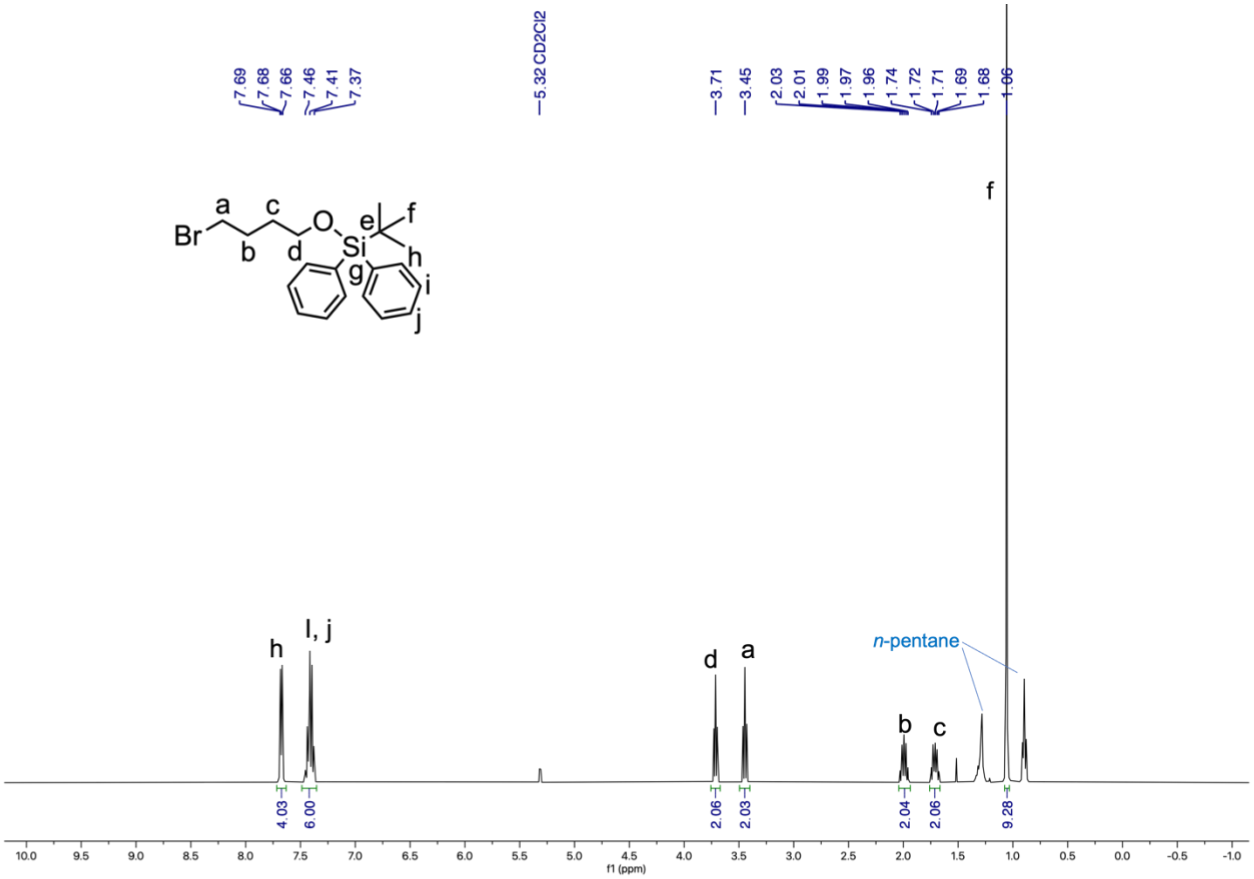

Fig. S3. 400 MHz ^{1}H NMR spectrum of 15 in CD_2Cl_2.

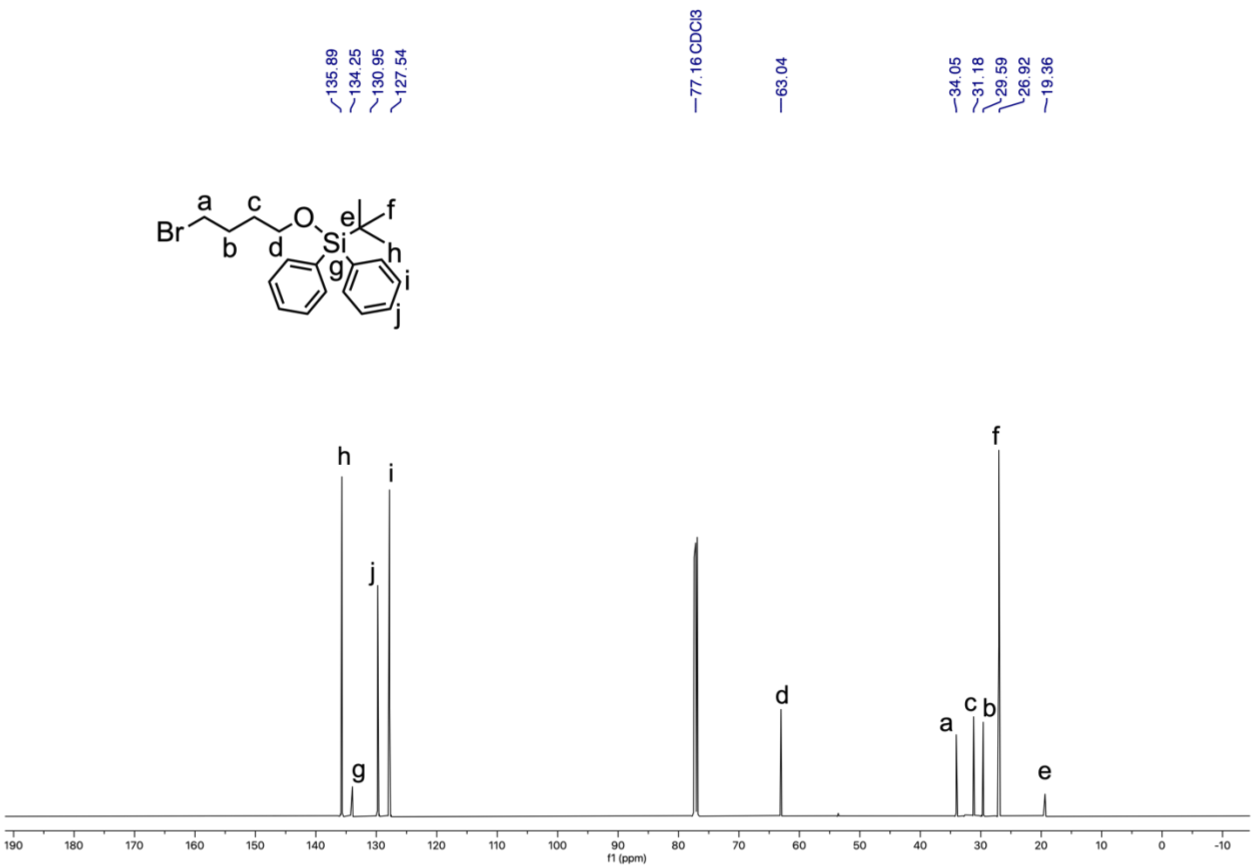

Fig. S4. 126 MHz ^{13}C NMR spectrum of 15 in $CDCl_3$.

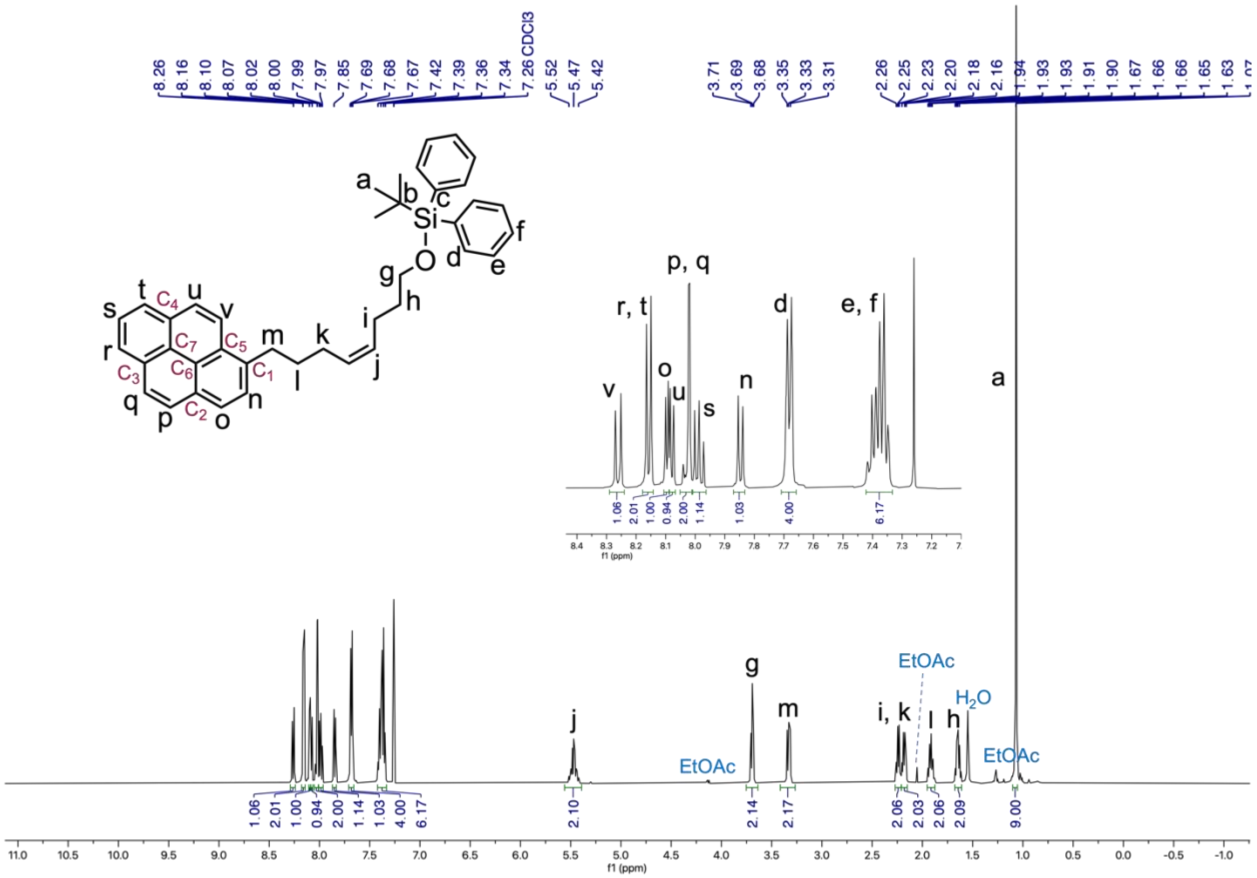

Fig. S5. 500 MHz ^{1}H NMR spectrum of 17 in $CDCl_3$.

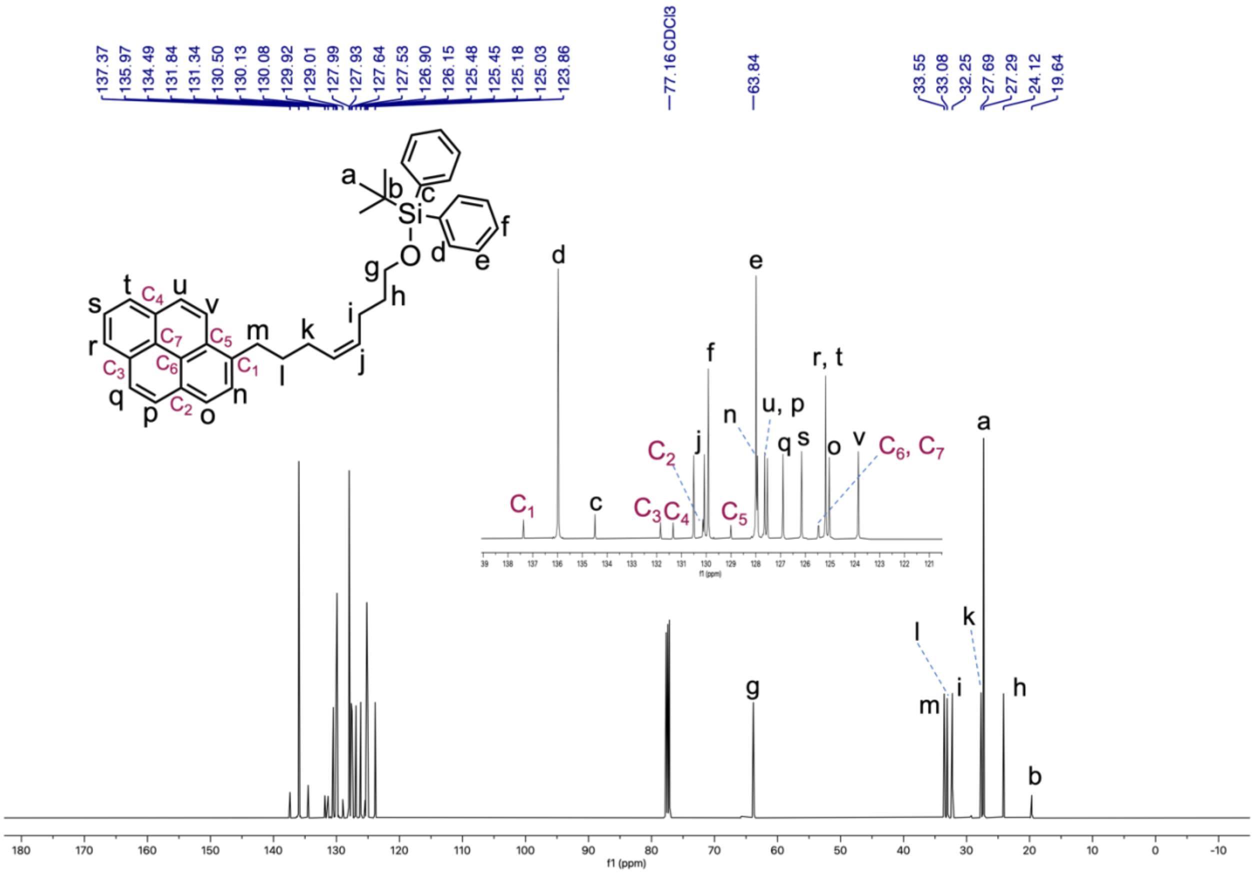

Fig. S6. 126 MHz ^{13}C NMR spectrum of 17 in $CDCl_3$.

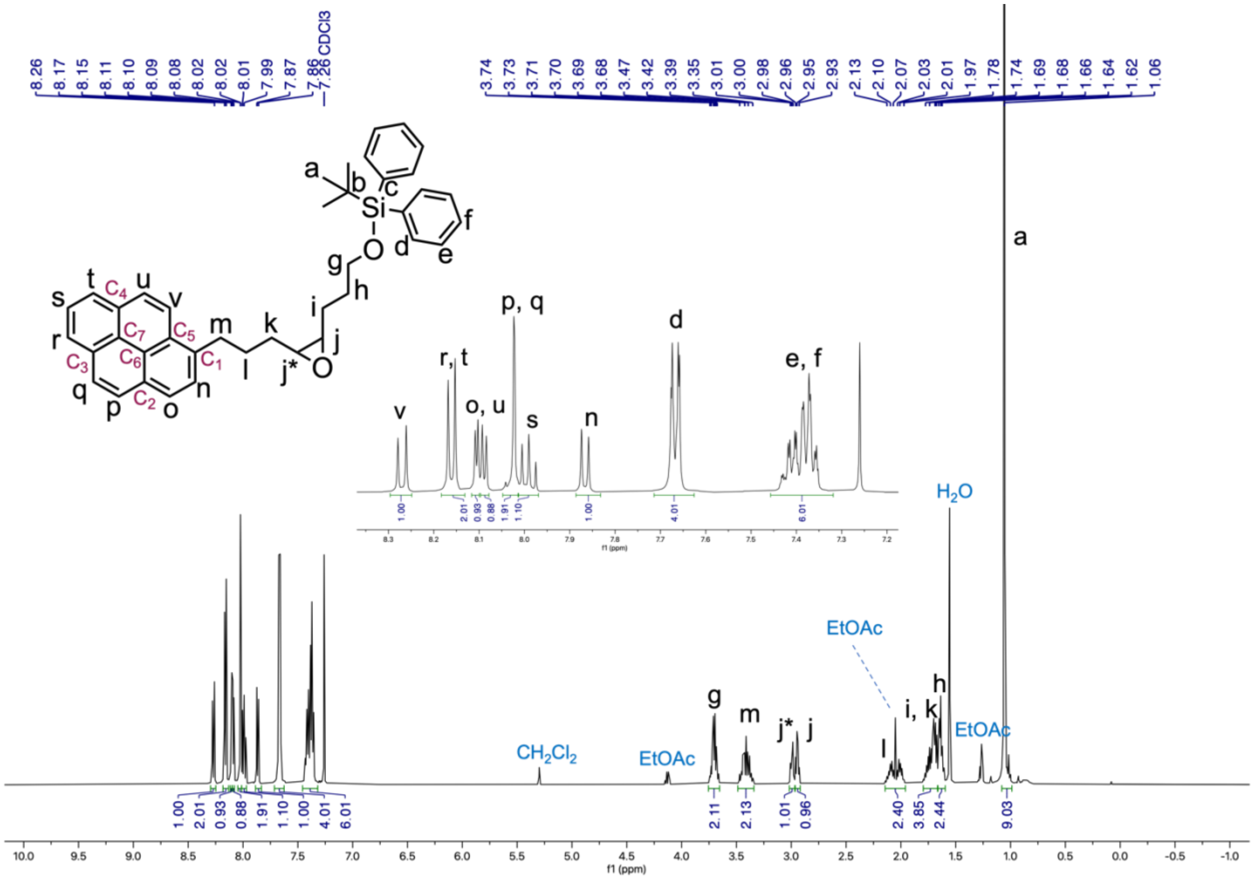

Fig. S7. 500 MHz ^{1}H NMR spectrum of 19 in $CDCl_3$.

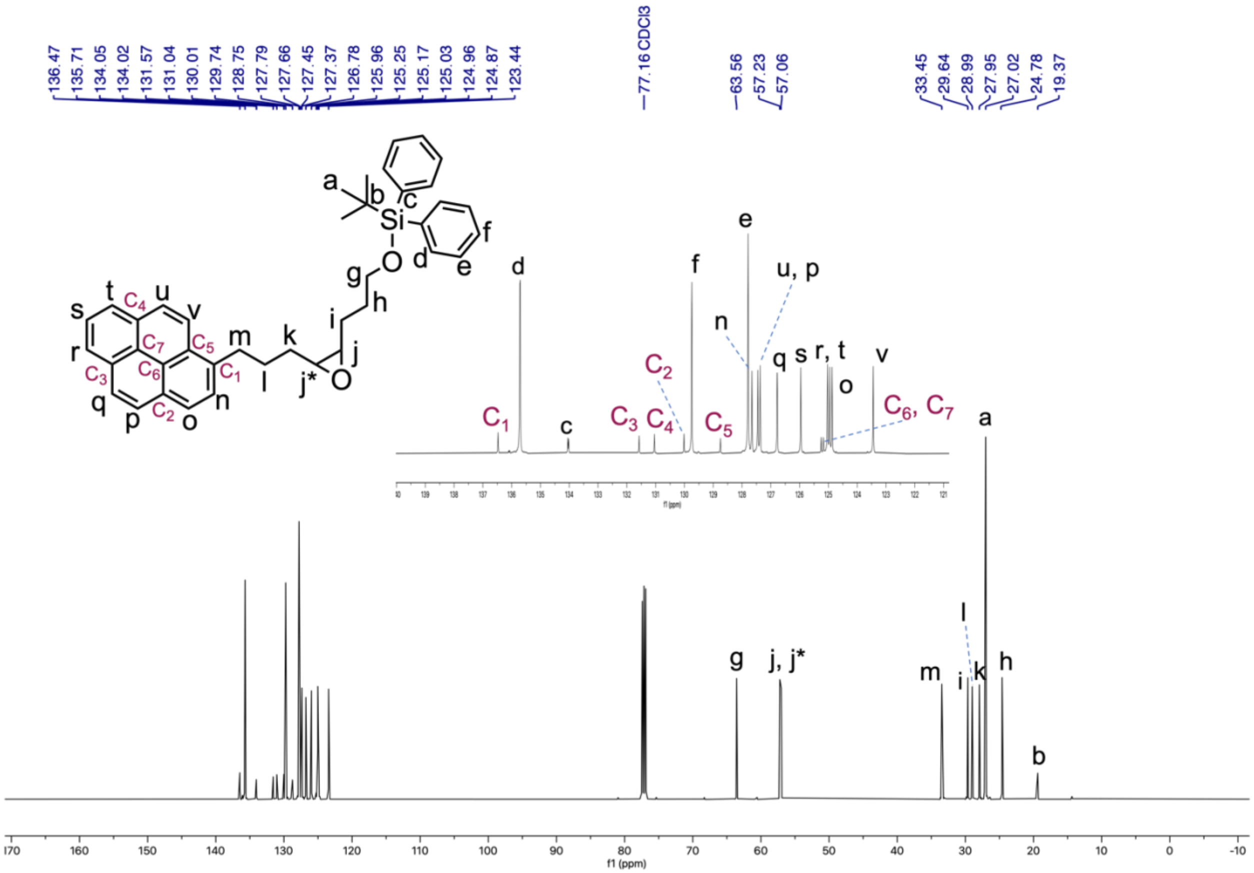

Fig. S8. 126 MHz ^{13}C NMR spectrum of 19 in $CDCl_3$.

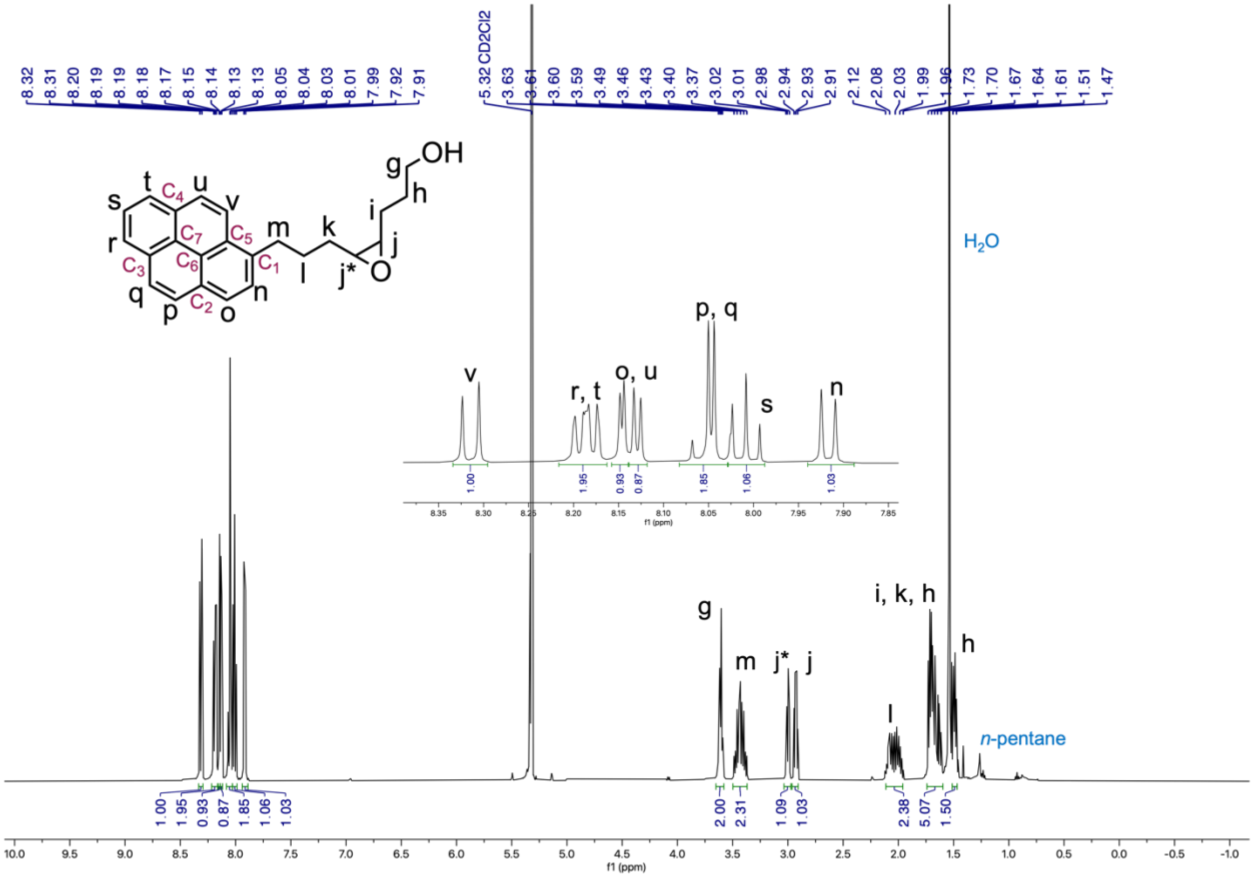

Fig. S9. 500 MHz ^{1}H NMR spectrum of 6 in CD_2Cl_2.

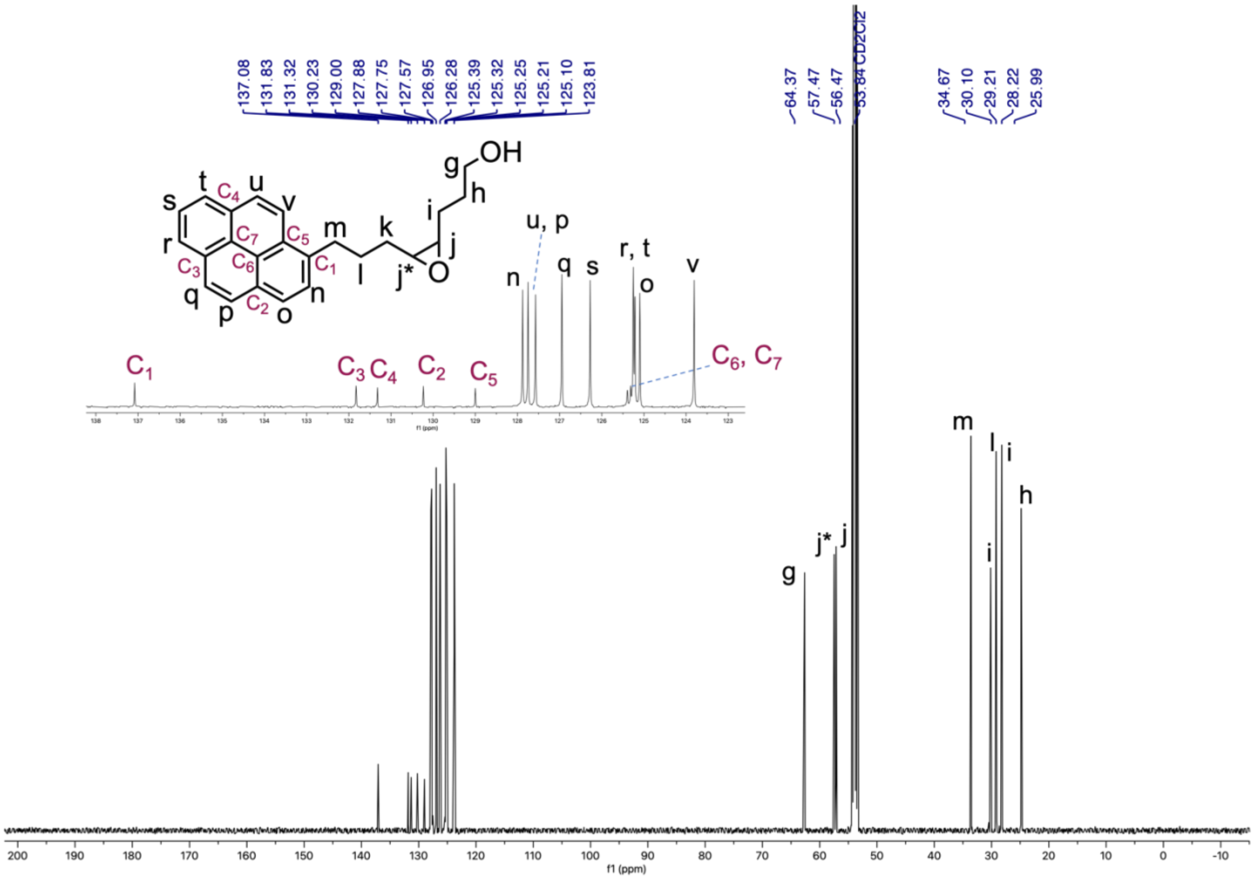

Fig. S10. 126 MHz ^{13}C NMR spectrum of 6 in CD_2Cl_2.

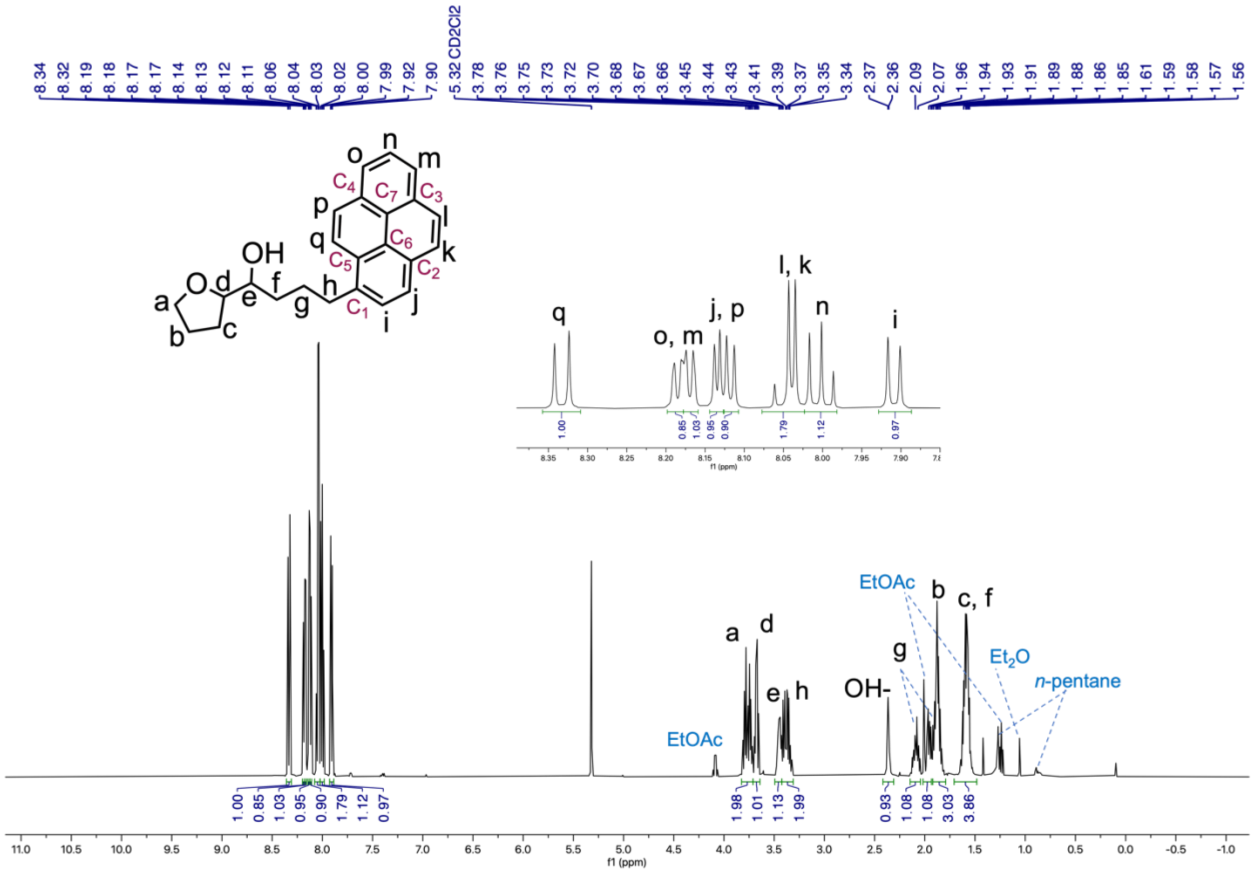

Fig. S11. 500 MHz ^{1}H NMR spectrum of 7 in CD_2Cl_2.

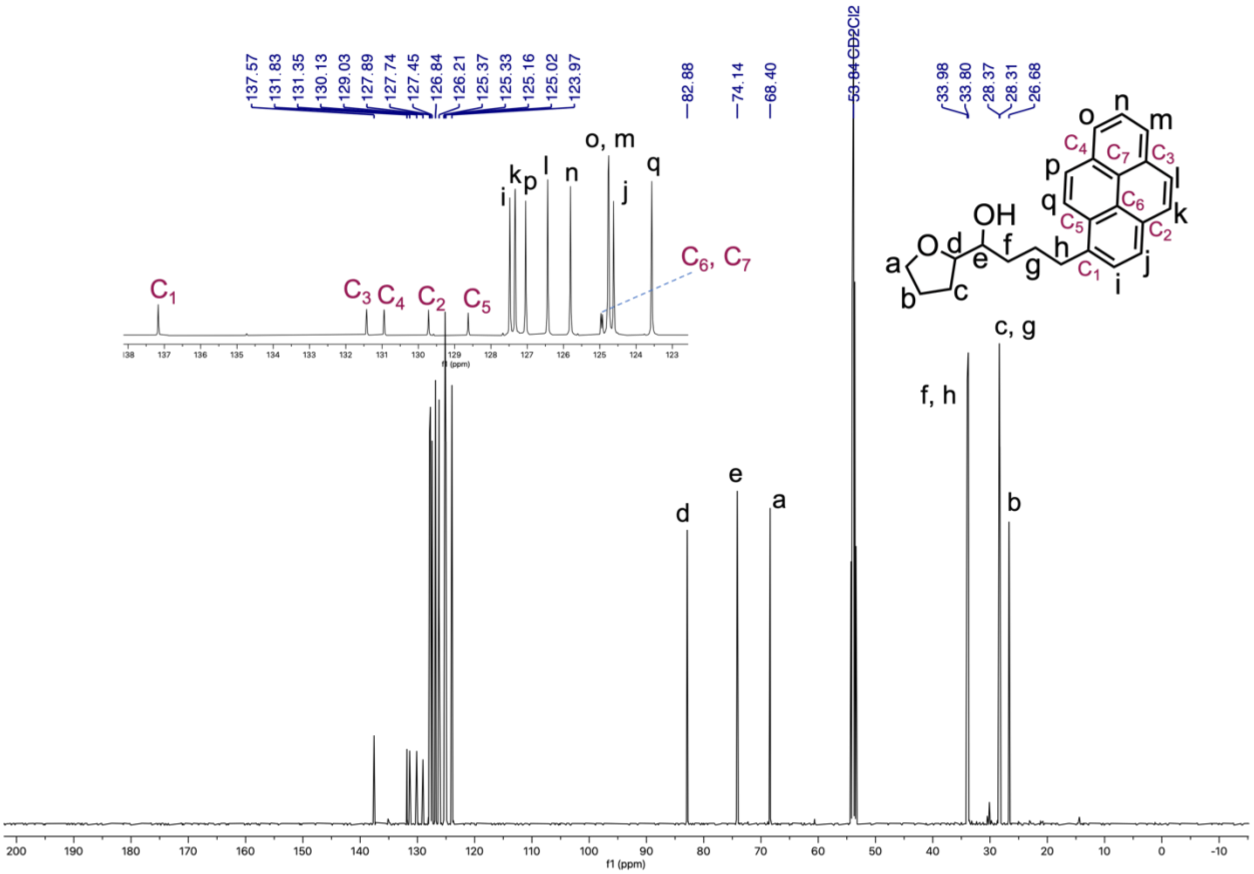

Fig. S12. 126 MHz ^{13}C NMR spectrum of 7 in CD_2Cl_2.

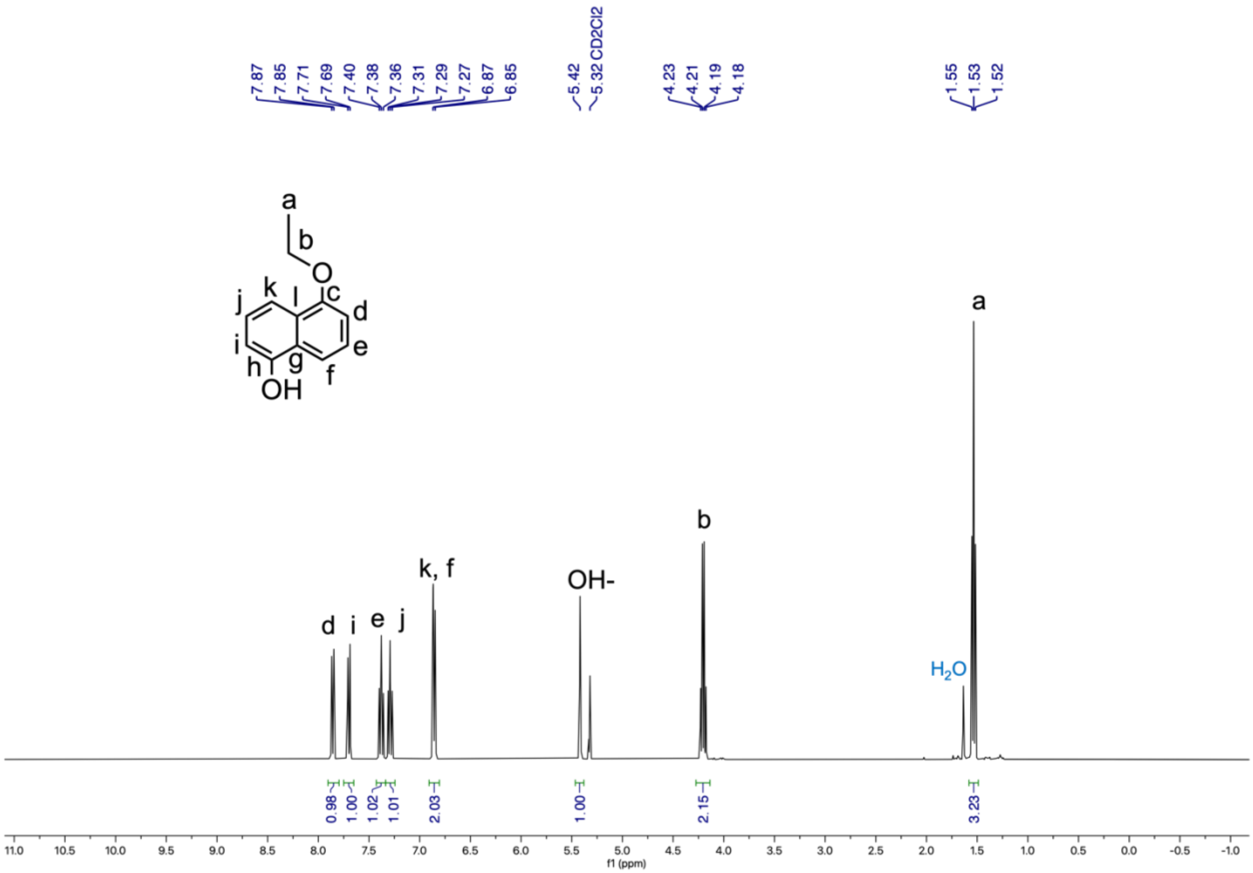

Fig. S13. 400 MHz ^{1}H NMR spectrum of 21 in CD_2Cl_2.

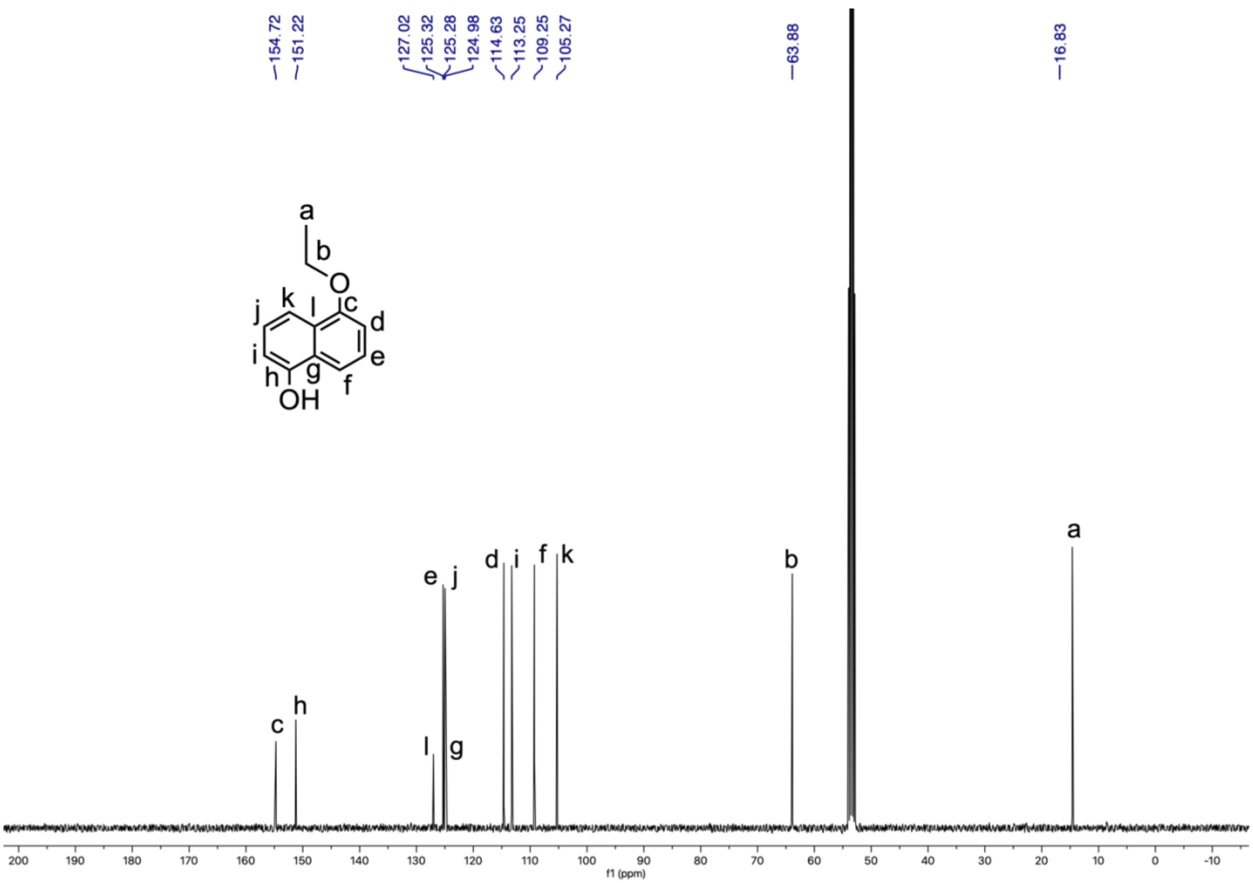

Fig. S14. 101 MHz ^{13}C NMR spectrum of 21 in CD_2Cl_2.

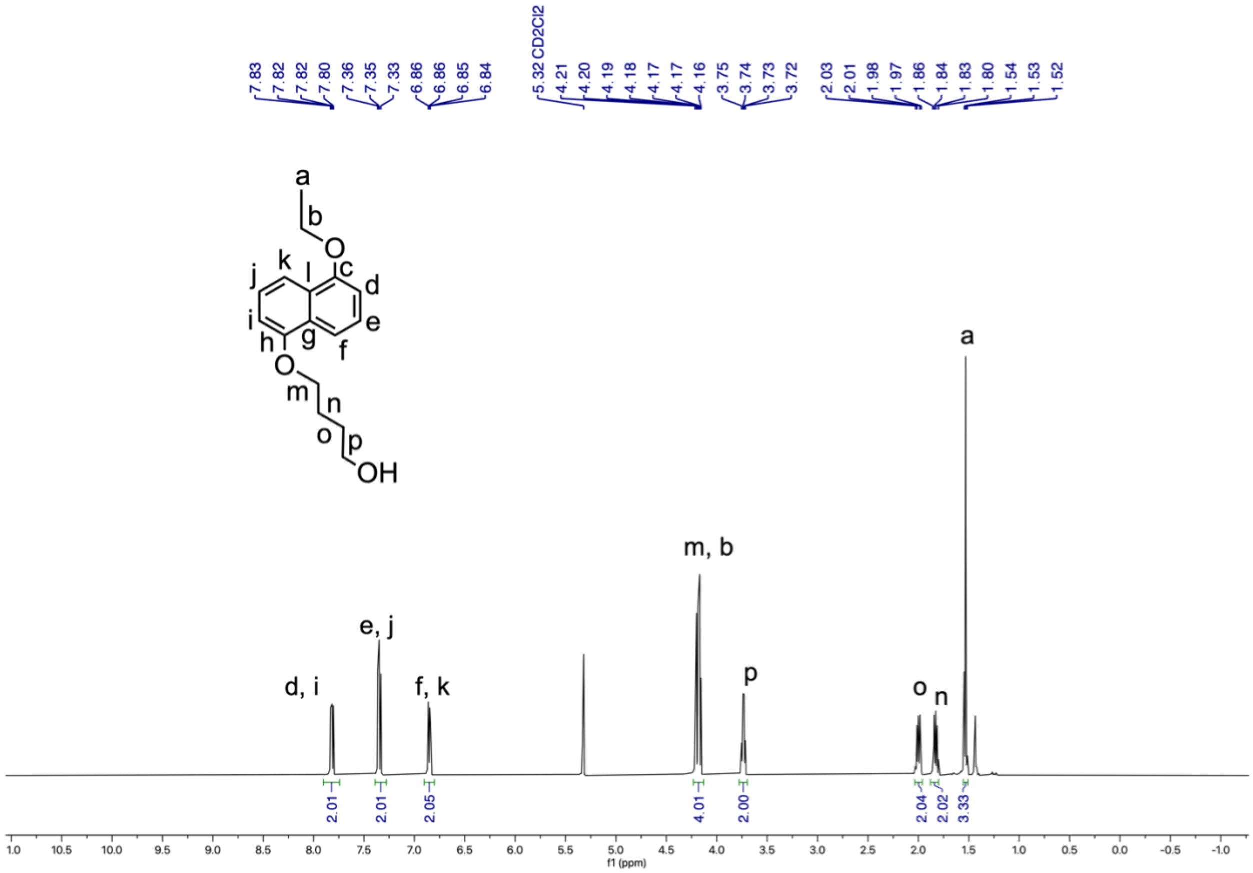

Fig. S15. 500 MHz ^{1}H NMR spectrum of 22 in CD_2Cl_2.

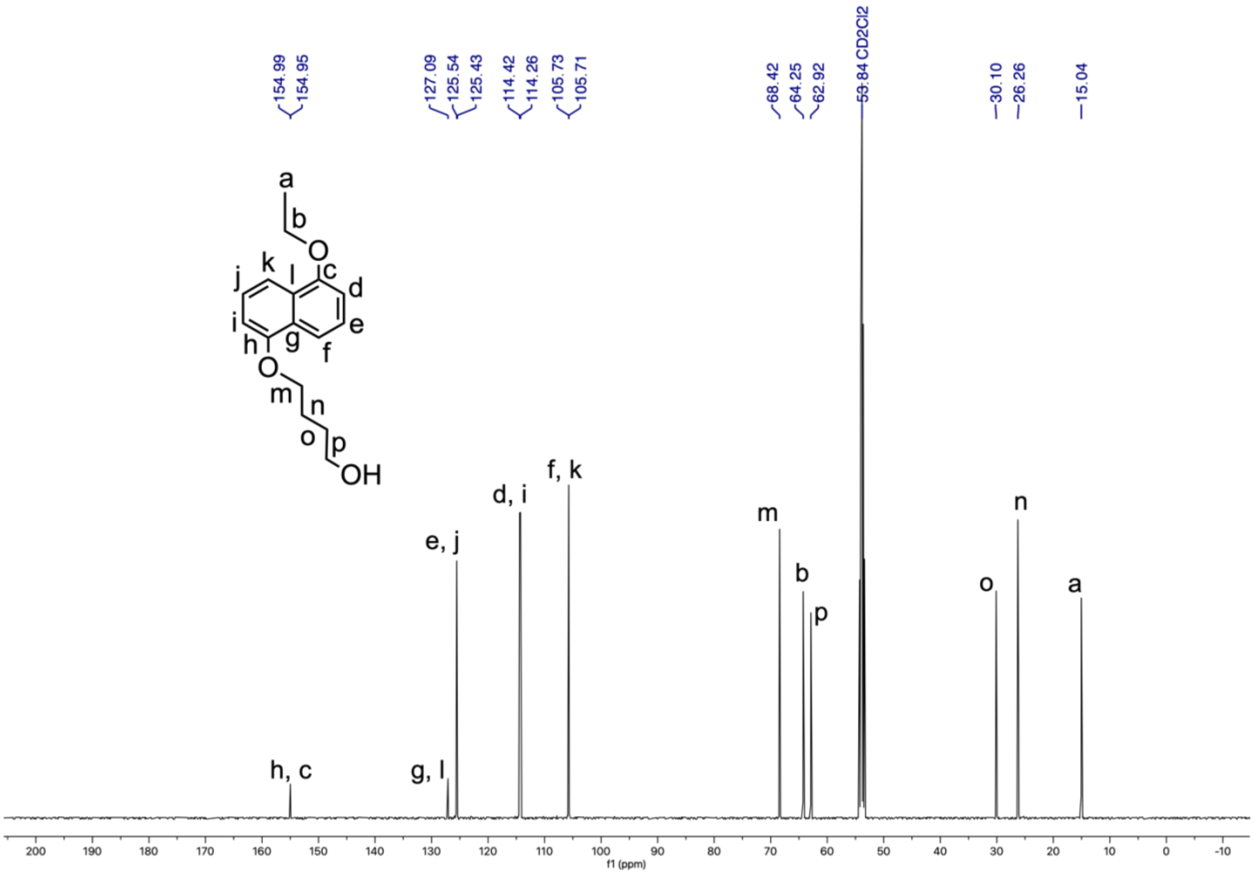

Fig. S16. 126 MHz ^{13}C NMR spectrum of 22 in CD_2Cl_2.

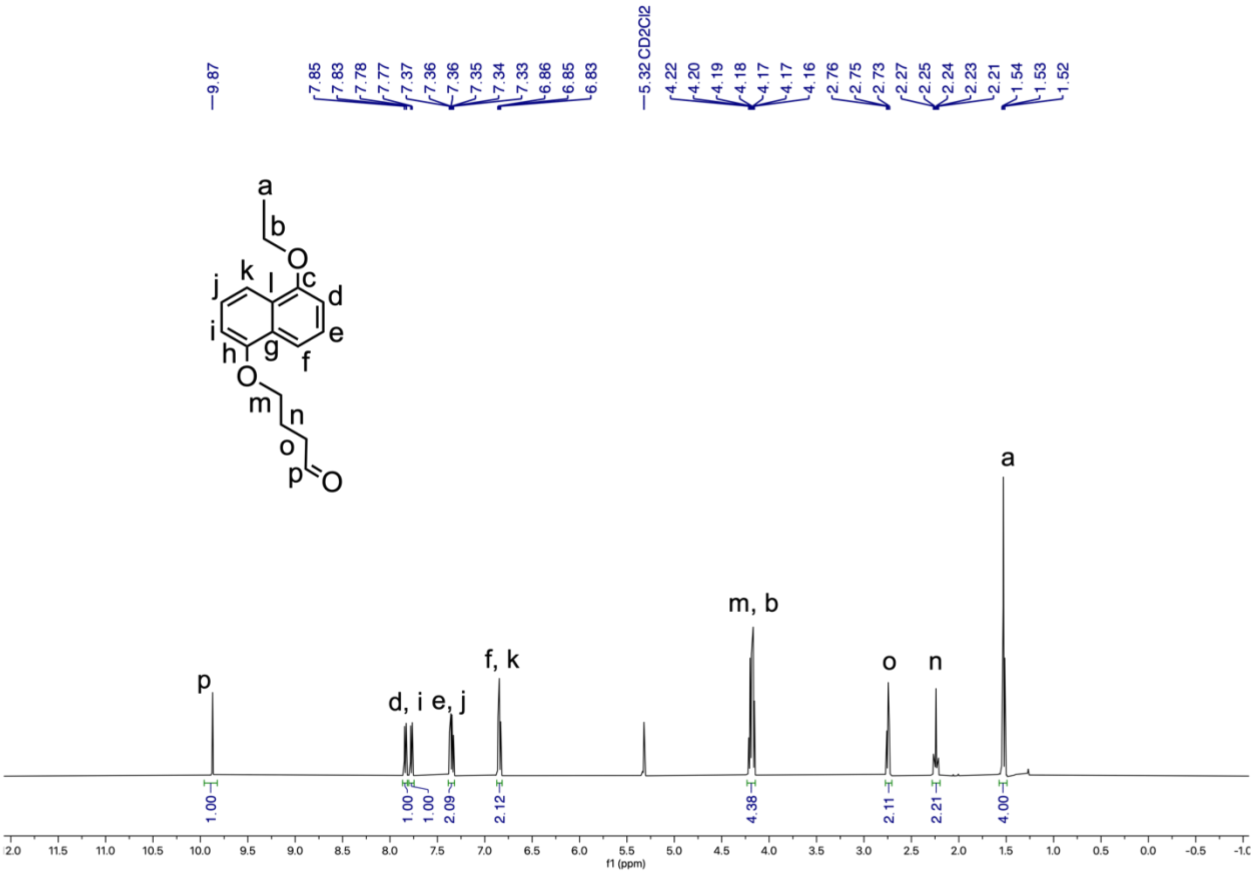

Fig. S17. 500 MHz ^{1}H NMR spectrum of 23 in CD_2Cl_2.

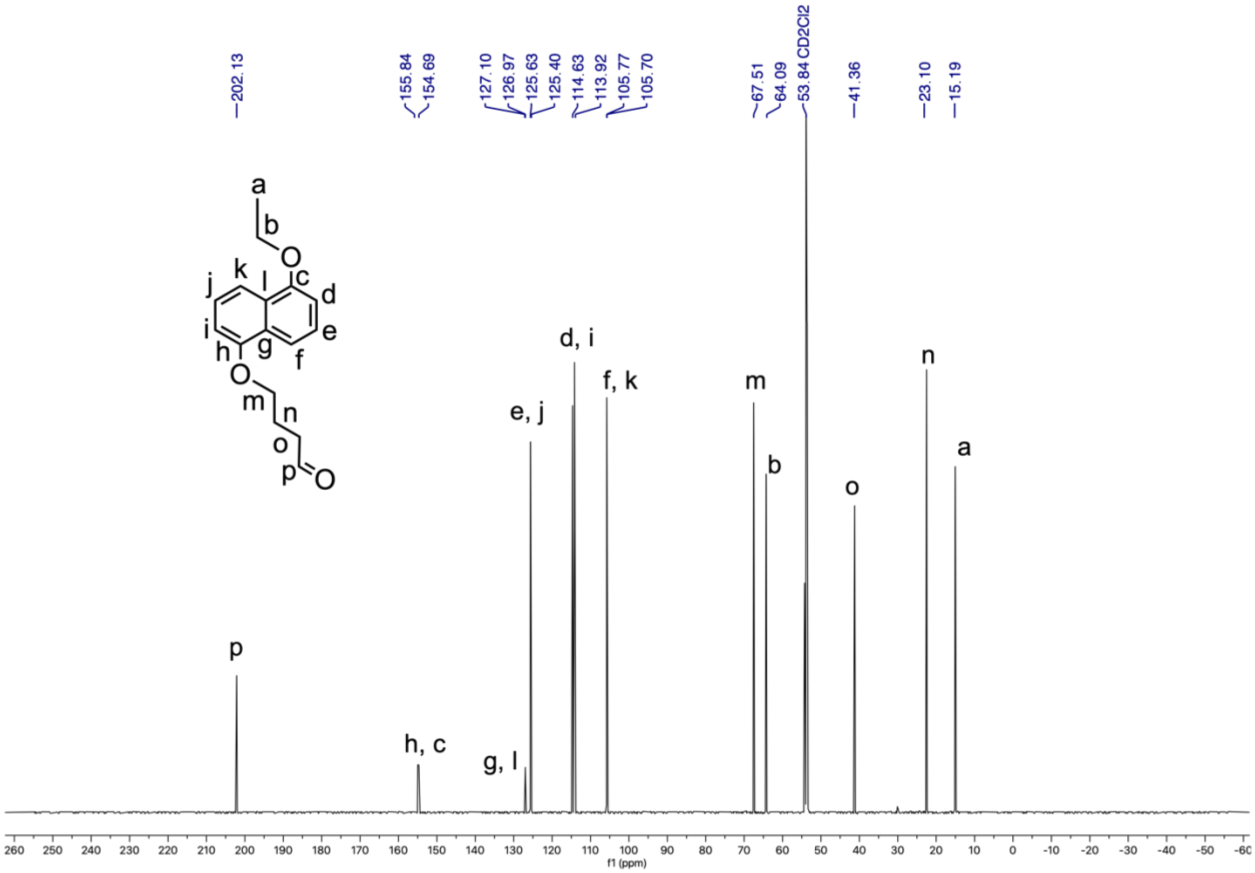

Fig. S18. 126 MHz ^{13}C NMR spectrum of 23 in CD_2Cl_2.

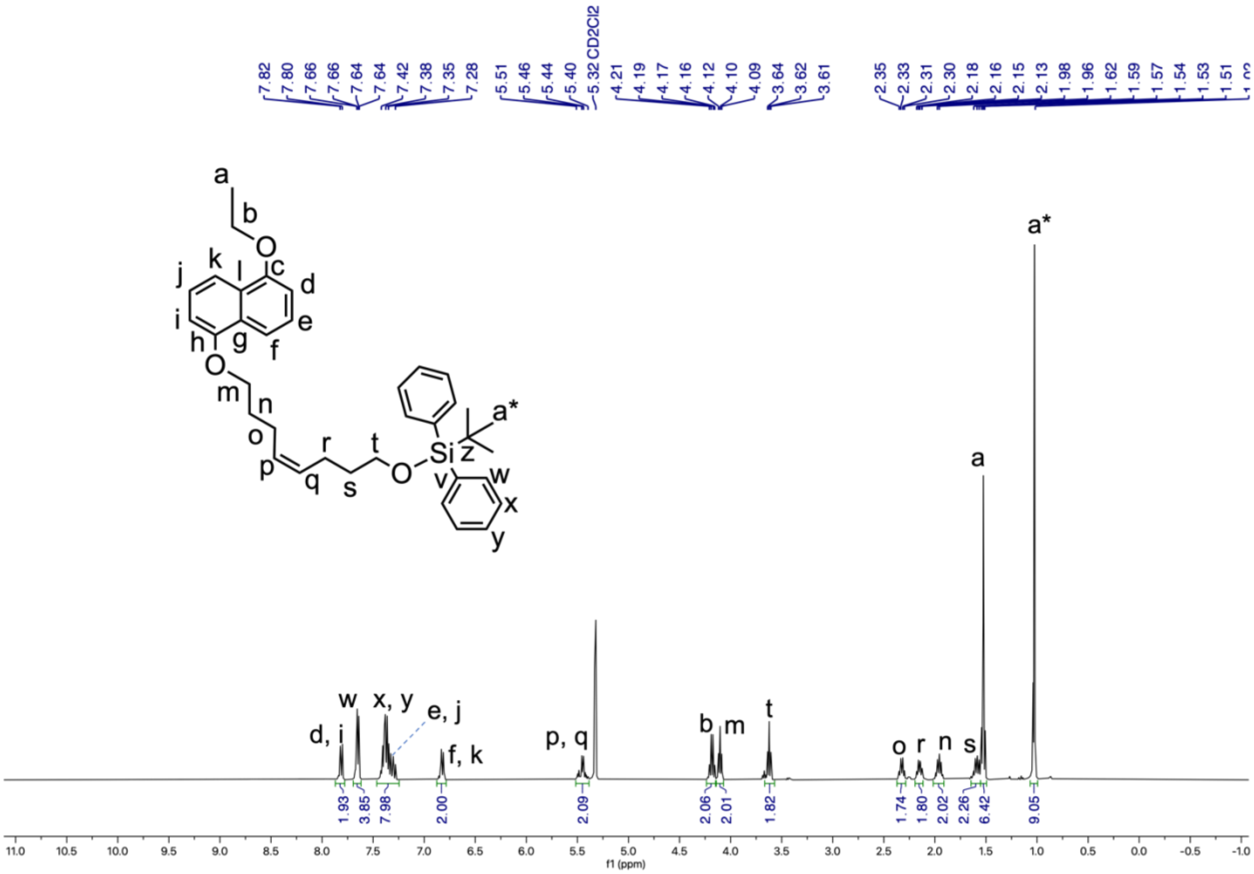

Fig. S19. 500 MHz ^{1}H NMR spectrum of 24 in CD_2Cl_2.

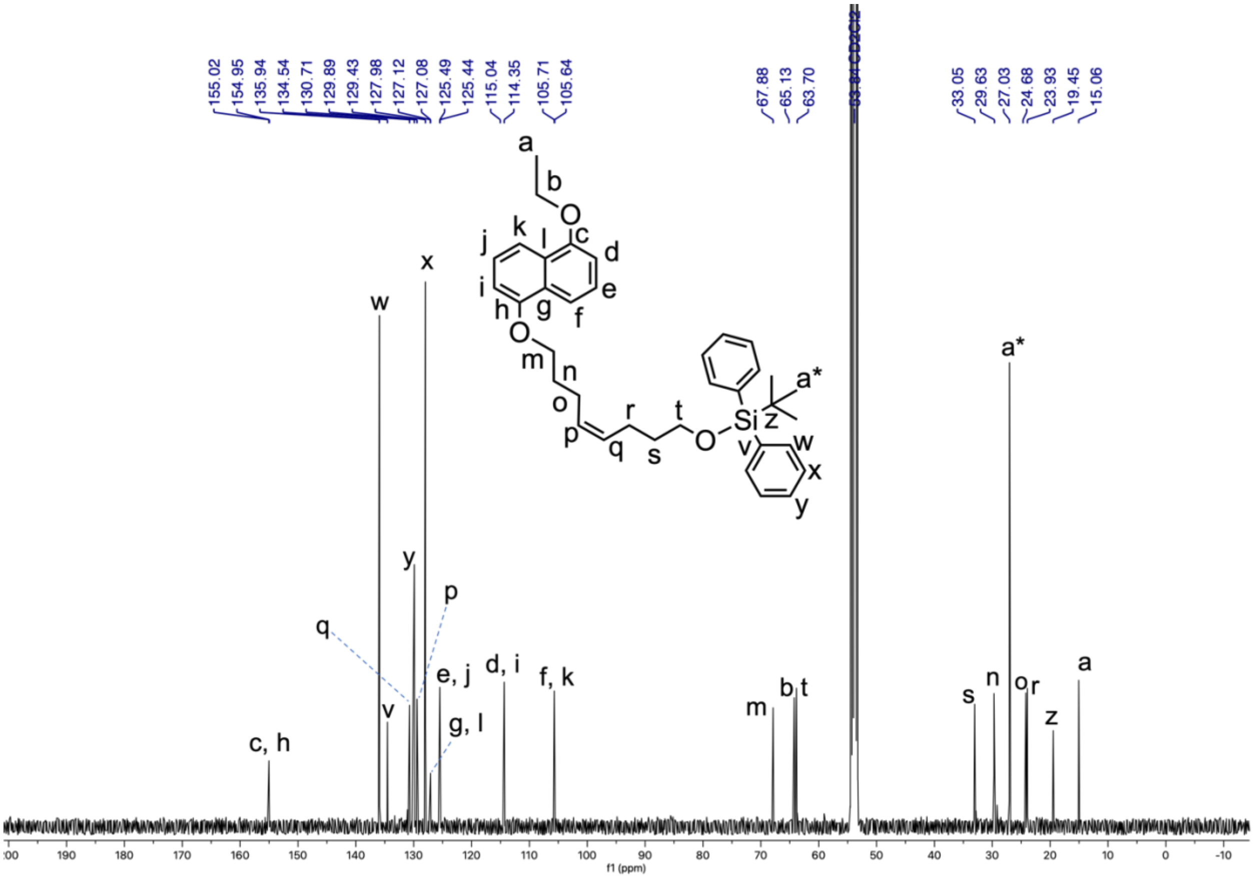

Fig. S20. 126 MHz ^{13}C NMR spectrum of 24 in CD_2Cl_2.

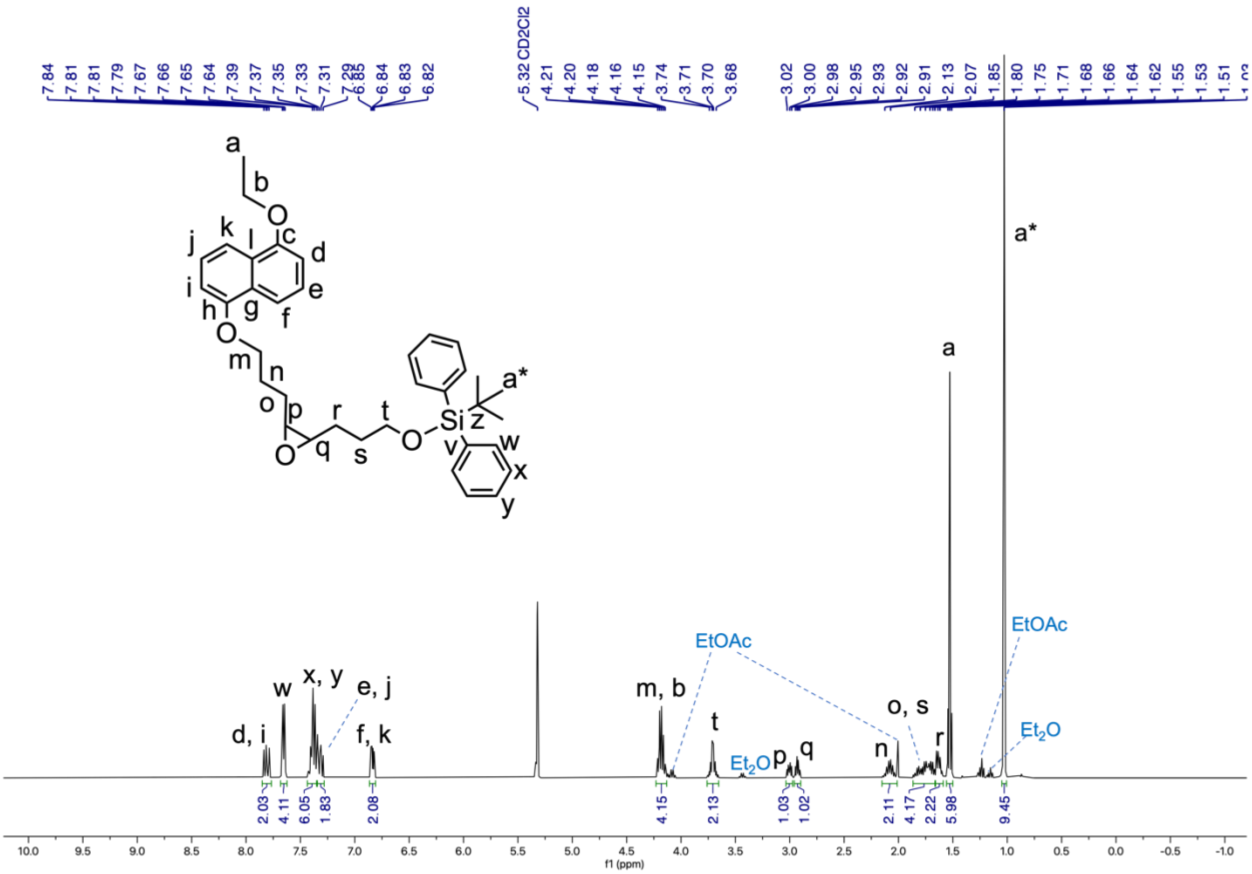

Fig. S21. 400 MHz ^{1}H NMR spectrum of 25 in CD_2Cl_2.

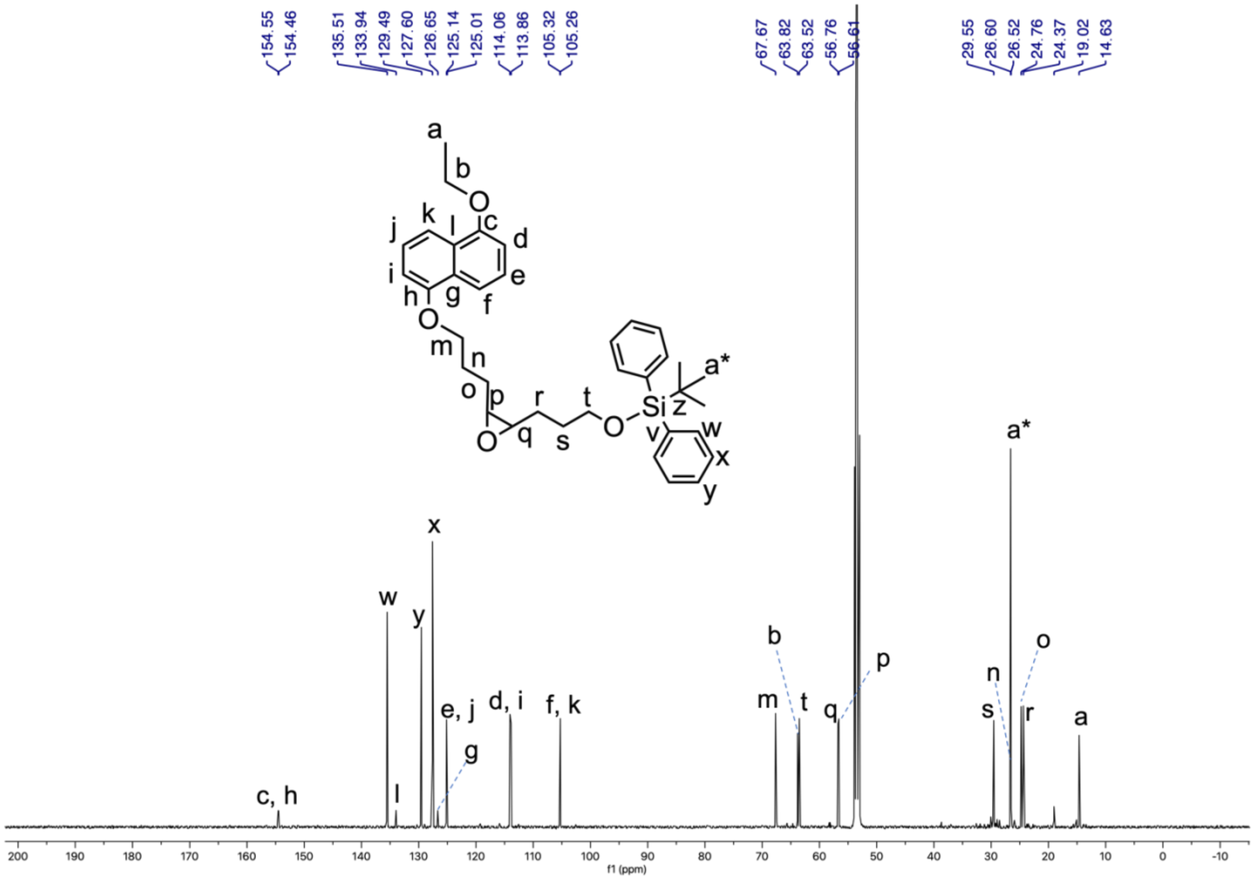

Fig. S22. 126 MHz ^{13}C NMR spectrum of 25 in CD_2Cl_2.

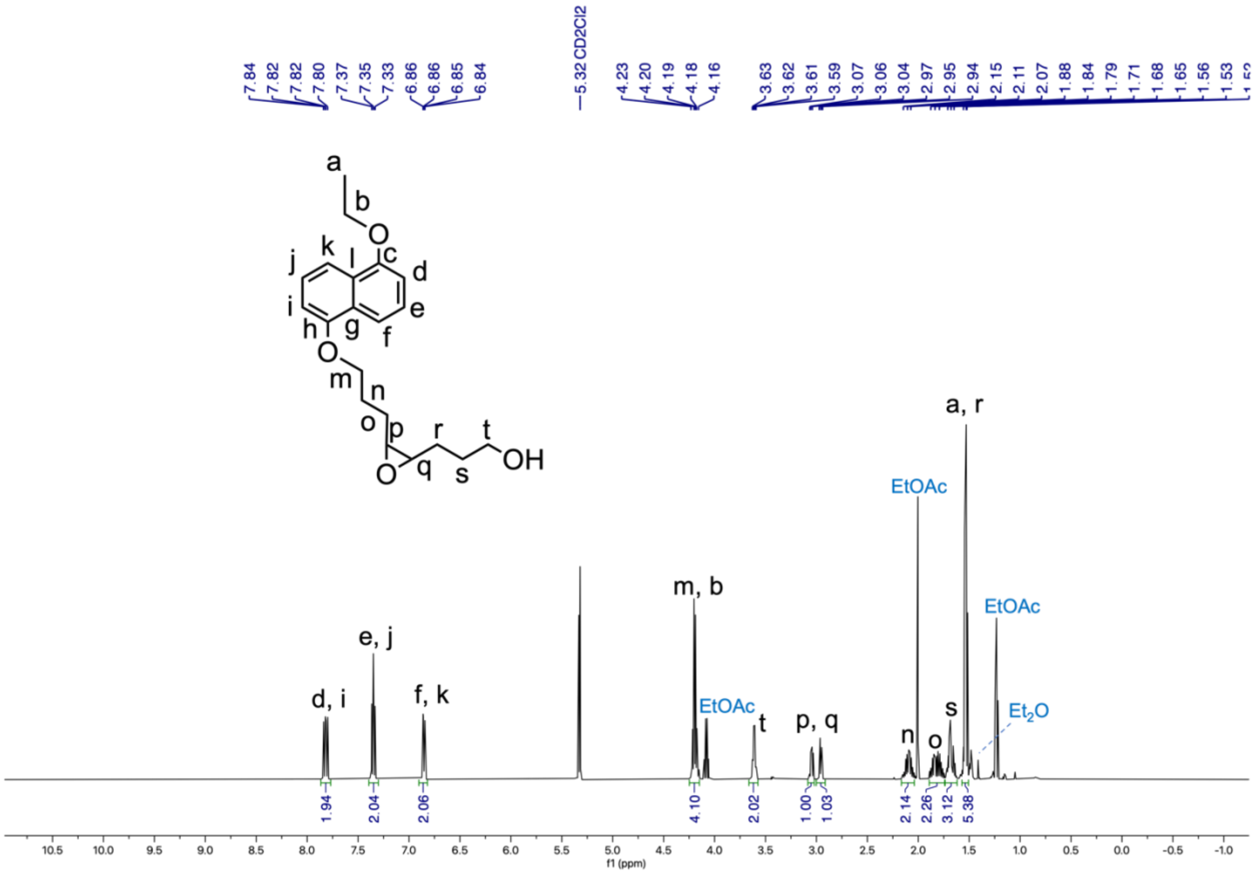

Fig. S23. 500 MHz ^{1}H NMR spectrum of 8 in CD_2Cl_2.

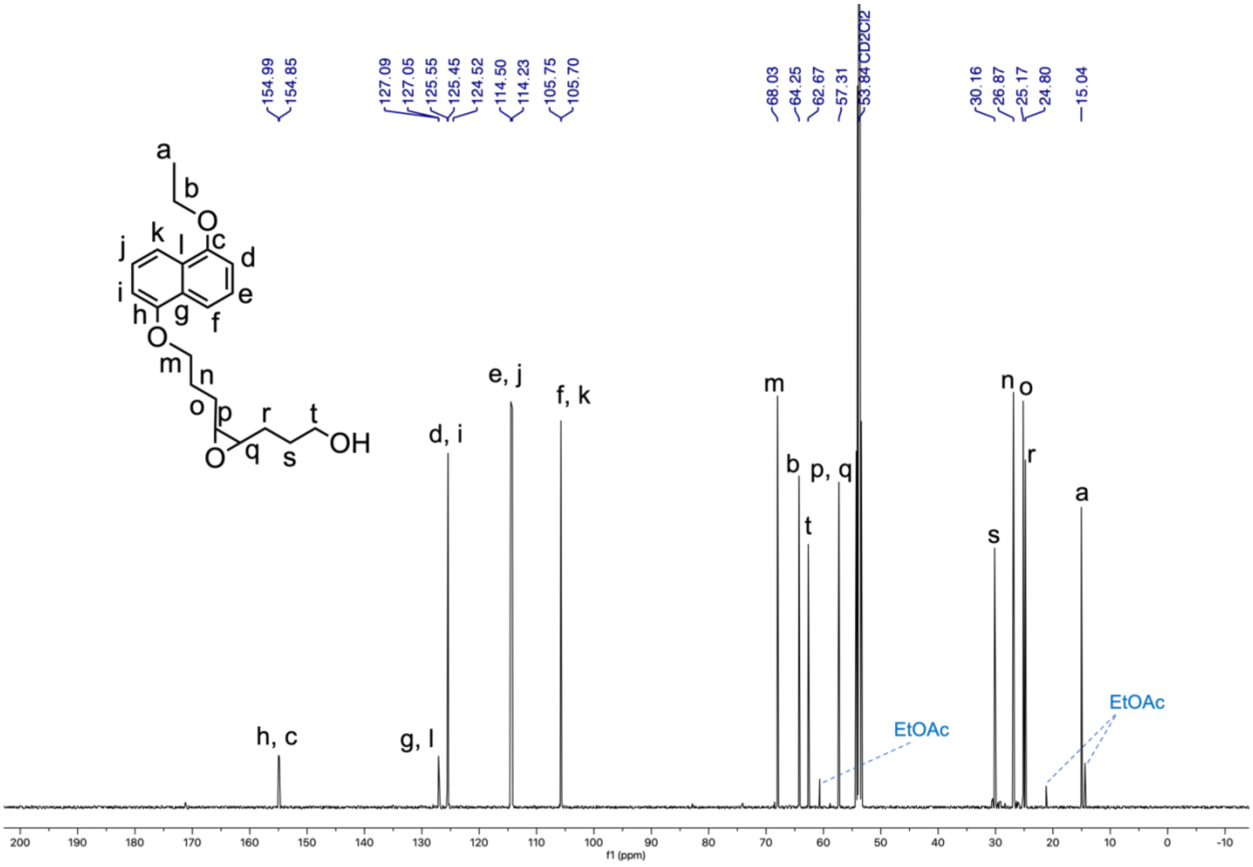

Fig. S24. 126 MHz ^{13}C NMR spectrum of 8 in CD_2Cl_2.

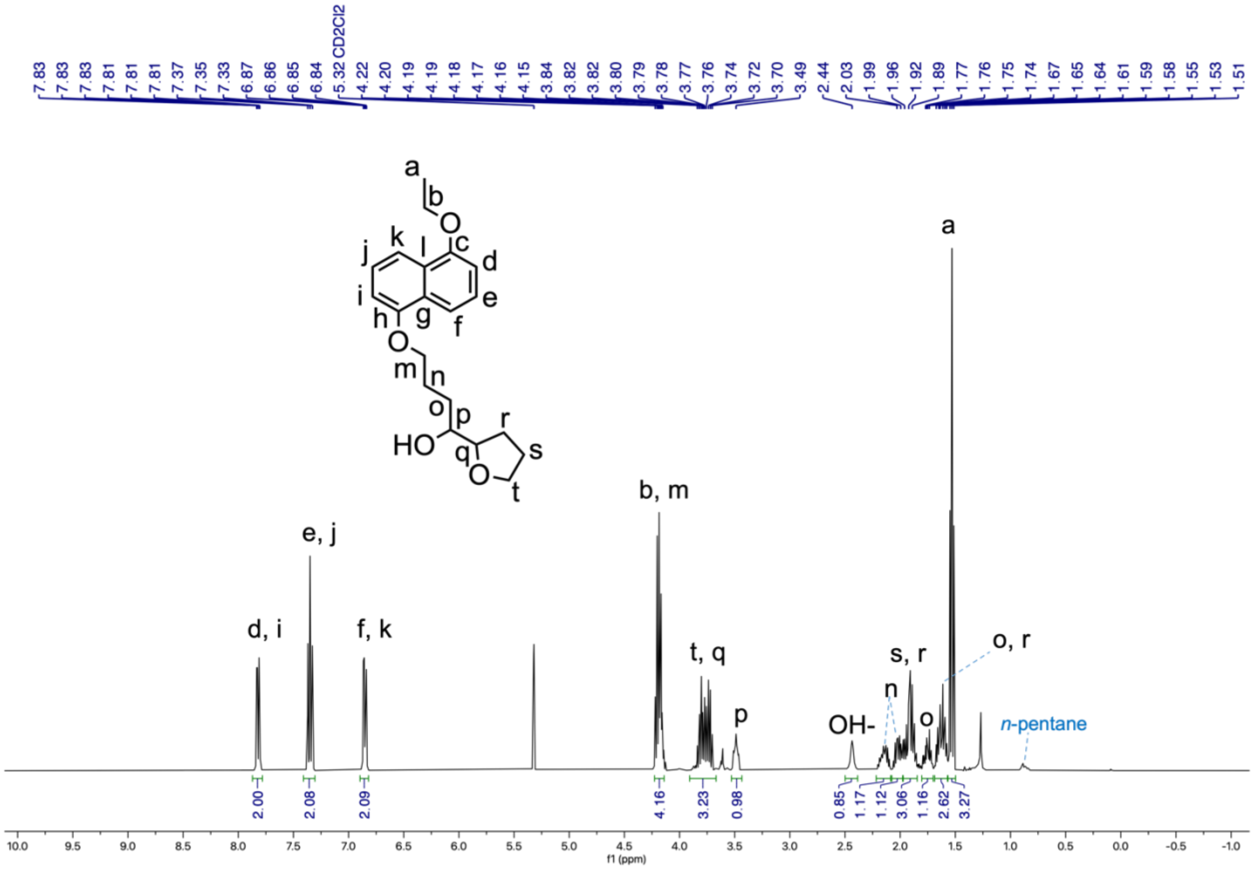

Fig. S25. 400 MHz ^{1}H NMR spectrum of 26 in CD_2Cl_2.

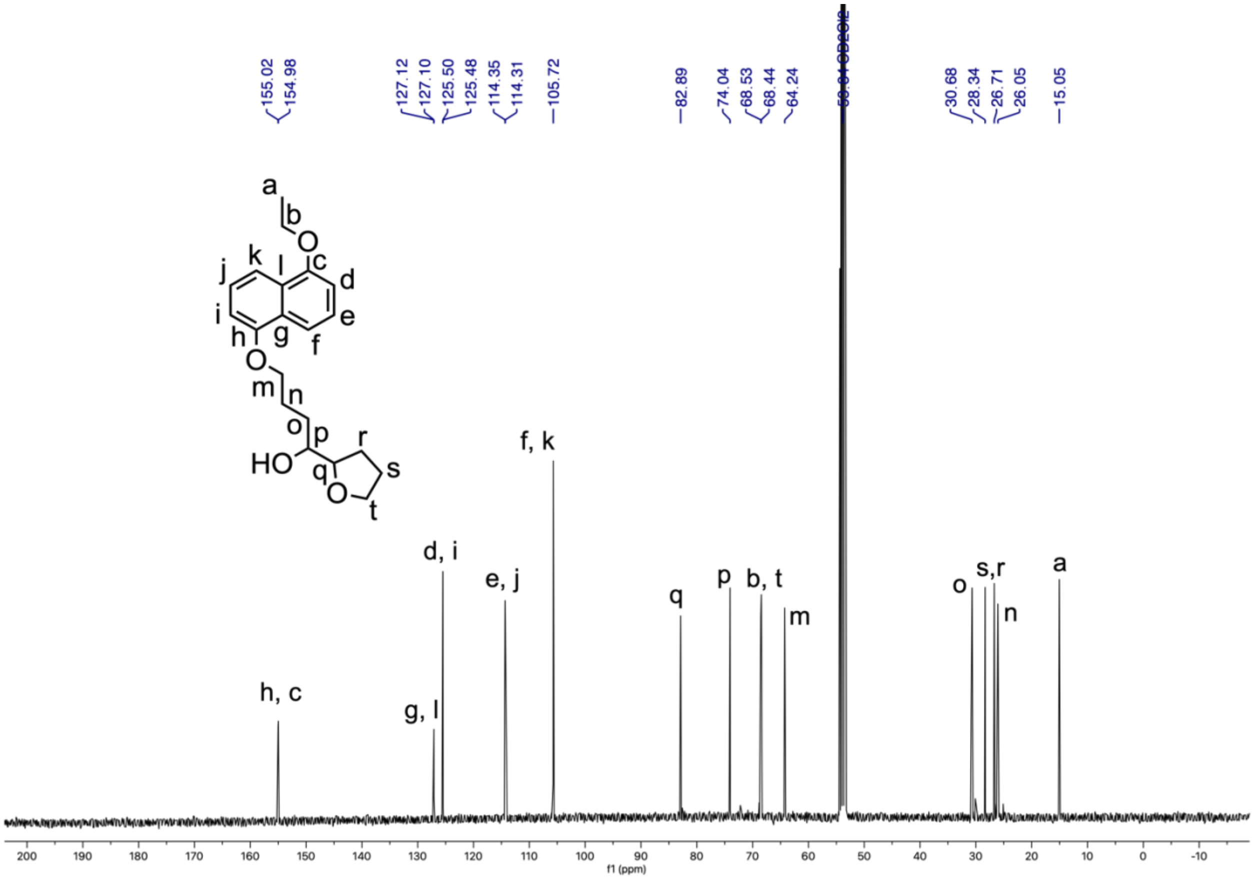

Fig. S26. 101 MHz ^{13}C NMR spectrum of 26 in CD_2Cl_2.

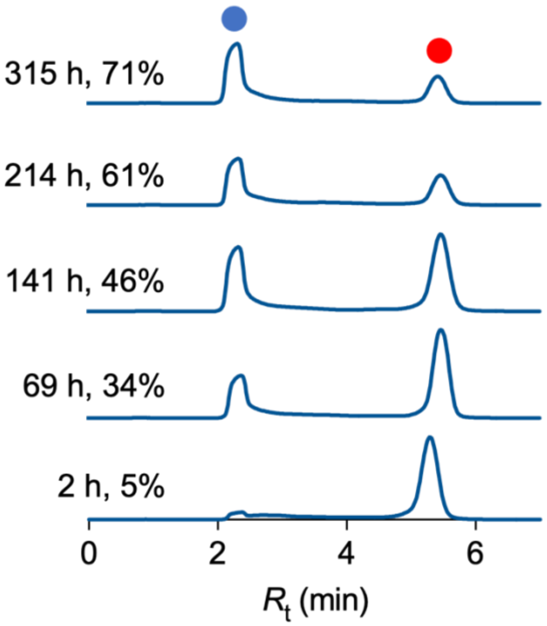

Fig. S27. HPLC analysis for 6.
Representative HPLC profile showing the time course of the cyclization of **6** (100 mM) in the presence of MWCNTs **2** (3% wt) in ODCB at 40 °C. Samples (1 drop ~ 5 µL) were taken in appropriate intervals and diluted in CH_2Cl_2. Peaks of substrate **6** (red) and product **7** (blue) were integrated to determine conversion. HPLC analysis: YMC Pack SIL 120 A, S-3 μm, size: 50 x 4 mm, 0.8 mL/min, 10% (EtOAc + 1% Et_3N) in CH_2Cl_2, λ_{abs} = 342 nm. R_t (**6**): 5.48 min and R_t (**7**): 2.40 min.

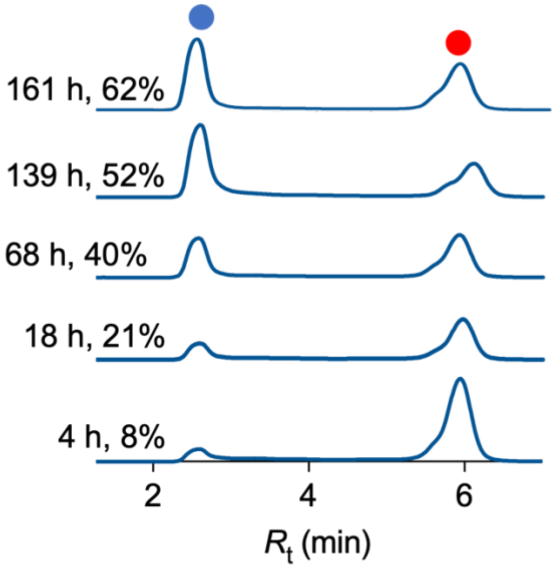

Fig. S28. HPLC analysis for 8.

Representative HPLC profile showing the time course of the cyclization of **8** (100 mM) in the presence of MWCNTs **2** (3% wt) in ODCB at 40 °C. Samples (1 drop ~ 5 μL) were taken in appropriate intervals and diluted in CH_2Cl_2. Peaks of substrate **8** (red) and product **26** (blue) were integrated to determine conversion. HPLC analysis: YMC Pack SIL 120 A, S-3 μm, size: 50 x 4 mm, 0.8 mL/min, 30% (EtOAc + 1% Et_3N) in *n*-hexane, λ_{abs} = 300 nm. R_t (**8**): 5.97 min and R_t (**26**): 2.58 min.

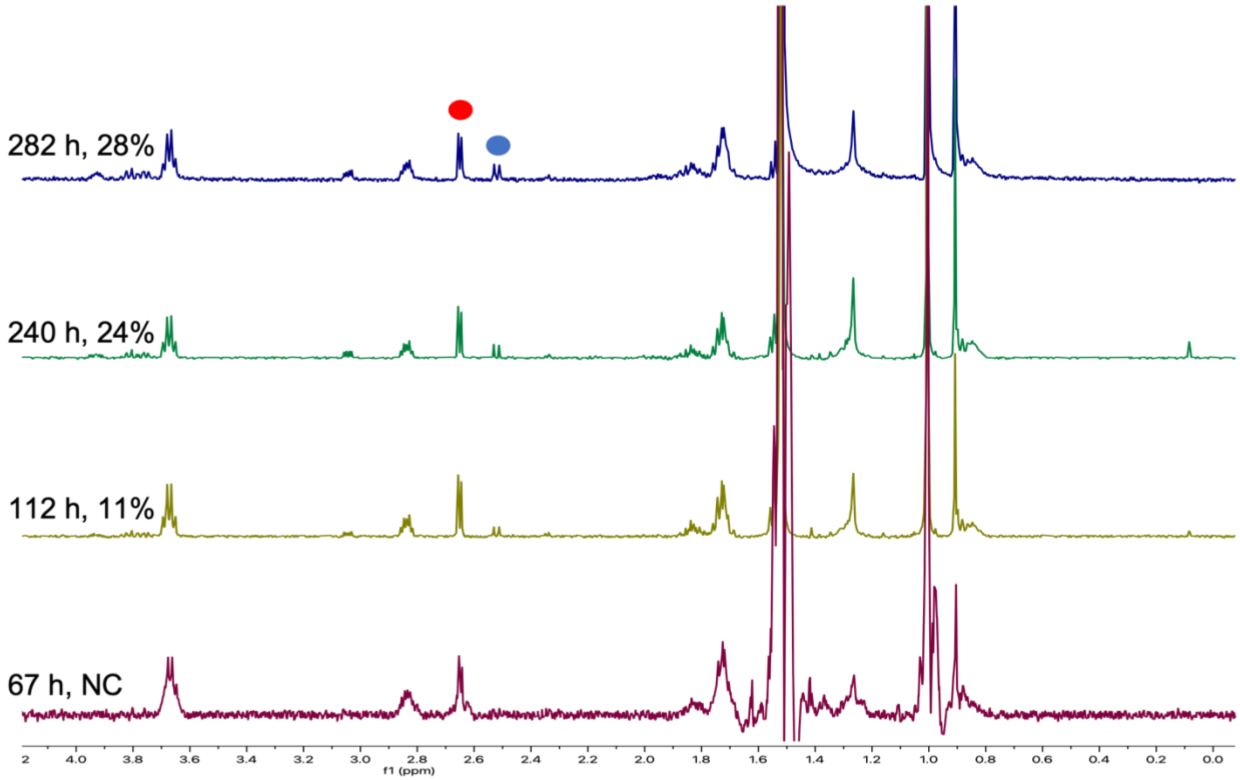

Fig. S29. ^{1}H NMR analysis for 9.
Representative ^{1}H NMR profile (400 MHz, CD_2Cl_2) showing the time course of the cyclization of **9** (100 mM) in the presence of MWCNTs **2** (3% wt) in ODCB at 60 °C. Samples (1 drop ~ 5 μL) were taken in appropriate intervals and diluted in CD_2Cl_2. Peaks of substrate **9** (red, 2.65 ppm) and product **27** (blue, 2.52 ppm) were integrated to determine the conversion. NC: No detectable conversion.

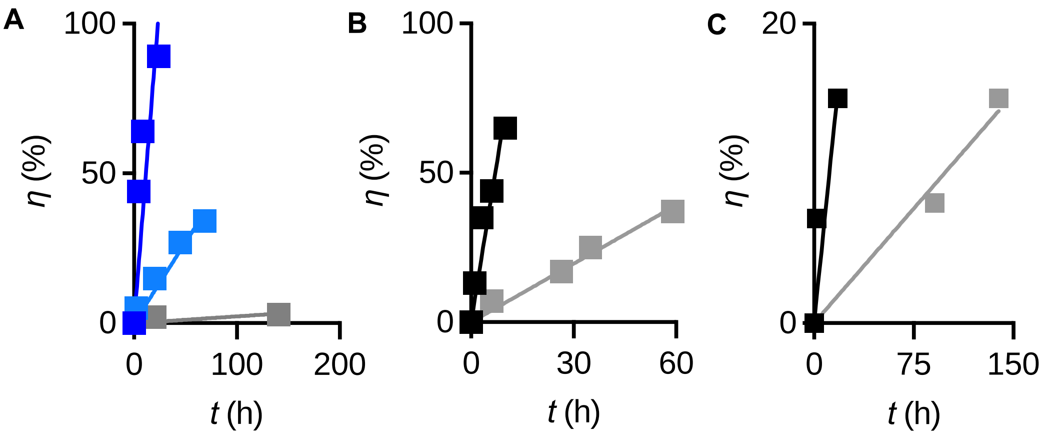

Fig. S30. Initial substrate 6 consumption with time in ODCB at 40 °C.
(A) Conversion of **6** with time in the presence of 0 (grey), 3 (blue), or 9 (dark blue) wt% MWCNTs **2**. (B) Conversion of **6** with time in the presence of 0 (black) or 25 (grey) mol% of **10** with 5 wt% MWCNTs **2**. (C) Conversion of **6** with time in the presence of 0 (black) or 25 (grey) mol% of **7** with 3 wt% MWCNTs **2**.

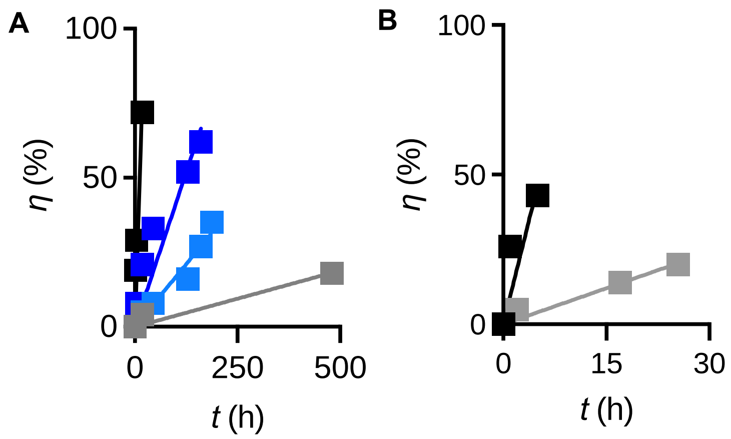

Fig. S31. Initial substrate 8 consumption with time in ODCB at 40 °C.
(A) Conversion of **8** with time in the presence of 0 (grey), 1 (blue), 3 (dark blue), or 9 (black) wt% MWCNTs **2**. (B) Conversion of **8** with time in the presence of 0 (black) or 25 (grey) mol% of **10** with 5 wt% MWCNTs **2**.

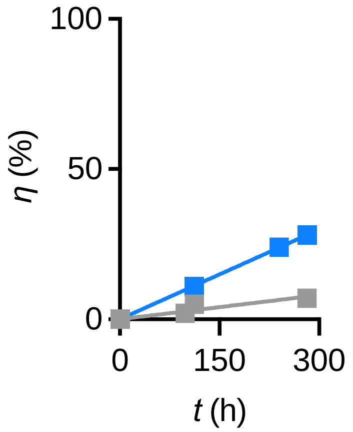

Fig. S32. Initial substrate 9 consumption in ODCB at 60 °C.
Conversion of **9** with time in the presence of 0 (grey) or 3 (blue) wt% MWCNTs **2.**

Table S1. Catalytic (k_{cat}) and inhibition (k_i) rate constants. 100 mM substrate S, 0-9 wt% MWCNT catalyst, ODCB at 40 or 60 °C (see Cond) with or without inhibitor I in mol% compared to S.

entry	S	MWCNT (wt%)	k_{cat} (h^{-1})	k_{uncat} (h^{-1})	k_i (h^{-1})	k_{cat}/k_{ref}	Cond	I, c (mol%)
1	**6**	3	5.4×10^{-4}	2.3×10^{-5}	-	24	40 °C	-
2	**6**	9	8.0×10^{-3}	2.3×10^{-5}	-	350	40 °C	-
3	**8**	1	1.7×10^{-3}	3.8×10^{-4}	-	4	40 °C	-
4	**8**	3	4.1×10^{-3}	3.8×10^{-4}	-	10	40 °C	-
5	**8**	9	4.2×10^{-2}	3.8×10^{-4}	-	110	40 °C	-
6	**9**	3	9.9×10^{-2}	2.7×10^{-2}	-	4	60 °C	-
7	**6**	5	7.1×10^{-3}	-	6.5×10^{-4}	11	40 °C	**10**, 25
8	**8**	5	9.3×10^{-3}	-	8.4×10^{-4}	12	40 °C	**10**, 25
9	**6**	3	5.4×10^{-4}	-	1.0×10^{-4}	5	40 °C	**7**, 25
